# Differential Effect of HCV Eradication and Fibrosis Grade on Hepatocellular Carcinoma and All-cause Mortality

**DOI:** 10.1038/s41598-018-31839-y

**Published:** 2018-09-12

**Authors:** Yun Bin Lee, Joon Yeul Nam, Jeong-Hoon Lee, Young Chang, Hyeki Cho, Young Youn Cho, Eun Ju Cho, Su Jong Yu, Hwi Young Kim, Dong Ho Lee, Jeong Min Lee, Seong Gyu Hwang, Yoon Jun Kim, Jung-Hwan Yoon

**Affiliations:** 1 Department of Internal Medicine and Liver Research Institute, Seoul National University College of Medicine, Seoul, Korea; 2 Department of Internal Medicine, CHA Bundang Medical Center, CHA University, Seongnam, Korea; 3 Department of Internal Medicine and Liver Center, Ewha Womans University School of Medicine, Seoul, Korea; 4 Department of Radiology, Seoul National University College of Medicine, Seoul, Korea

**Keywords:** Hepatitis C, Hepatocellular carcinoma

## Abstract

Whether a sustained virological response (SVR) improves long-term outcomes in chronic hepatitis C patients with earlier-stage fibrosis has not been established. We investigated the differential effect of SVR on the risk of outcomes according to hepatic fibrosis grade. Fibrosis grade was categorised using FIB-4: <1.45, low-probability of significant fibrosis; 1.45–3.25, intermediate-probability; and ≥3.25, high-probability. Primary and secondary endpoints were hepatocellular carcinoma (HCC) occurrence and death, respectively. Among 1,373 included chronic hepatitis C patients, 744 patients were treated with interferon-based or –free regimens and 622 (83.6%) achieved SVR. SVR was independently associated with lower risk of HCC (*vs*. untreated: adjusted hazard ratio [aHR], 0.165; 95% confidence interval [CI], 0.077–0.350; *P* < 0.001) and overall death (*vs*. untreated; aHR, 0.146; 95% CI, 0.050–0.424; *P* < 0.001) during the median observation of 3.5 (interquartile range, 1.9–6.6) years. The SVR group had significantly lower risk of HCC than the untreated group among patients with intermediate-probability (*n* = 492: aHR, 0.171; 95% CI, 0.051–0.578; *P* = 0.004) and high-probability (*n* = 446: aHR, 0.243; 95% CI, 0.107–0.551; *P* < 0.001) of significant fibrosis. HRs were maintained after balancing with inverse probability weighting. SVR was associated with reduced risk of HCC development and all-cause mortality in patients with chronic hepatitis C.

## Introduction

Chronic hepatitis C virus (HCV) infection is one of the major causes of chronic liver diseases such as cirrhosis, hepatic decompensation, and hepatocellular carcinoma (HCC)^1^. The goal of antiviral treatment of chronic hepatitis C (CHC) is eradication of HCV to prevent fibrosis progression and the development of liver-related and non-liver-related complications, leading to prolonged survival^2–4^. In a meta-analysis, HCV eradication by interferon (IFN)-based antiviral treatment was shown to directly reduce HCC risk^5^. The introduction of new direct-acting antivirals (DAAs) for the treatment of CHC has derived excellent sustained virological response (SVR) rates in most HCV-infected patients, including patients with advanced fibrosis and extrahepatic comorbidities. Current international guidelines recommend antiviral treatment for all patients with HCV-related chronic liver disease, except those with limited life expectancy owing to non-liver-related comorbidities^2–4^. Although the earlier studies demonstrated that DAA-induced SVR has little or no impact on the risk of developing HCC and even reported early occurrence and recurrence of HCC in virologically cured patients with DAA-based treatments^6–10^, recent studies using the US Veterans Administration database determined the significant association between DAA-induced SVR and reduction in the risk of HCC^11,12^. Moreover, SVR was associated with a reduced risk of HCC irrespective of type of antiviral treatments (either IFN-based therapy or DAA-based therapy)^12^.

Patients with advanced hepatic fibrosis are at increased risk of developing hepatic decompensation or HCC within a relatively short timeframe^13,14^, and the risk can be substantially reduced by successful HCV eradication^15–17^. Therefore, antiviral therapies must be initiated immediately in these patients if not contraindicated^2–4^. Although numerous studies have found the clinical benefits of antiviral therapy in patients with advanced liver disease^5,15,18–20^, direct evidence supporting antiviral treatment initiation at earlier stages of fibrosis is scarce. While the risk of developing hepatic decompensation, HCC, or death has been reported to be significantly lower in patients with METAVIR fibrosis grades F0–F2^13,14^, higher SVR rates are achievable and liver disease progression can be halted after SVR achievement in patients with lower-stage fibrosis, augmenting treatment benefits^2^. In this study, we aimed to analyse the association between eradication of HCV and the risk of HCC development and all-cause mortality in HCV-infected patients according to fibrosis stage of liver.

## Results

### Study population

Among 1,373 patients with chronic HCV infection included in the final analyses, 629 patients were not treated (the untreated group), 122 received antiviral treatment but failed to achieve SVR (the non-SVR group), and remaining 622 were treated and achieved SVR (the SVR group). Among 744 treated patients, 418 patients were treated with IFN-based therapy and 326 with DAAs. Patients treated with DAAs received daclatasvir/asunaprevir (*n* = 198) or elbasvir/grazoprevir (*n* = 4) for patients infected by genotype 1B HCV, sofosbuvir/ledipasvir (*n* = 39) or daclatasvir/sofosbuvir (*n* = 9) for patients infected by genotype 1 A HCV and genotype 1B HCV with resistance-associated variants (i.e., L31W/V/F/N/I/S/P/R and Y93N/H/C/P/D variants), or sofosbuvir plus ribavirin (*n* = 76) for patients infected by genotype 2 HCV. The median duration of follow-up was 3.5 (interquartile range [IQR], 1.9–6.6) years: 4.2 (IQR, 2.0–7.4) years in the untreated group, 5.6 (IQR, 3.1–7.4) years in the non-SVR group, and 2.5 (IQR, 1.8–5.8) years in the SVR group. Table 1 shows the baseline demographic and clinical characteristics of the study patients by group. The median age was 58 (IQR, 51–67) years overall, and was not significantly different between the groups (*P* = 0.33). Male patients accounted for 40.5%, and almost all patients were infected with HCV genotype 1 or 2. Prothrombin time was significantly longer in the non-SVR group and HCV viral load differed significantly between the groups (*P* = 0.03 and *P* < 0.001, respectively). While platelet count and serum ALT level differed between the groups, the differences were not statistically significant (*P* = 0.09 and *P* = 0.07, respectively).

**Table 1:** Baseline characteristics by group.

Characteristics	Overall	Untreated	Treated without SVR	Treated with SVR	*P* value
	(*N* = 1373)	(*n* = 629)	(*n* = 122)	(*n* = 622)	
Age, median (IQR), y	58 (51–67)	59 (51–67)	58 (50–65)	58 (50–68)	0.33
Male	556 (40.5)	258 (41.0)	52 (42.6)	246 (39.5)	0.77
Genotype (*n* = 1344)					0.10
1	742 (55.2)	346 (57.1)	70 (58.3)	326 (52.8)	
2	582 (43.3)	250 (41.3)	46 (38.3)	286 (46.3)	
Other	20 (1.5)	10 (1.7)	4 (3.3)	6 (1.0)	
Laboratory data, median (IQR)					
Platelet count, × 10^9^/L (*n* = 1204)	166 (127–208)	173 (128–214)	153 (114–207)	165 (128–203)	0.09
Albumin, g/dL (*n* = 1239)	4.2 (4.0–4.4)	4.2 (4.0–4.4)	4.1 (3.9–4.3)	4.2 (4.0–4.4)	0.76
Total bilirubin, mg/dL *(n* = 1239)	0.8 (0.6–1.1)	0.8 (0.6–1.1)	0.9 (0.7–1.1)	0.8 (0.7–1.1)	0.69
ALT, IU/L (*n* = 1242)	52 (28–97)	46 (27–82)	56 (30–97)	59 (29–107)	0.07
AST, IU/L (*n* = 1242)	53 (33–83)	48 (31–77)	54 (34–80)	56 (34–92)	0.12
GGT, U/L (*n* = 986)	42 (24–78)	43 (24–80)	50 (29–77)	40 (23–77)	0.17
International normalised ratio (*n* = 973)	1.03 (0.98–1.09)	1.04 (0.98–1.09)	1.05 (0.99–1.12)	1.02 (0.97–1.08)	0.03
AFP, ng/mL (*n* = 1343)	5.0 (3.2–9.0)	5.0 (3.0–9.0)	5.1 (3.8–10.0)	5.0 (3.4–8.6)	0.70
HCV RNA, log_10_ IU/mL (*n* = 1272)	6.03 (5.37–6.43)	5.98 (5.29–6.37)	6.41 (6.03–6.65)	6.03 (5.34–6.43)	<0.001
APRI (*n* = 1196)					0.27
<0.5	357 (29.8)	150 (32.9)	30 (24.6)	177 (28.6)	
≥0.5 to <1.5	519 (43.4)	193 (42.3)	60 (49.2)	266 (43.0)	
≥1.5	320 (26.8)	113 (24.8)	32 (26.2)	175 (28.3)	
FIB-4 (*n* = 1196)					0.63
<1.45	258 (21.6)	108 (23.7)	26 (21.3)	124 (20.1)	
≥1.45 to <3.25	492 (41.1)	185 (40.6)	47 (38.5)	260 (42.1)	
≥3.25	446 (37.3)	163 (35.7)	49 (40.2)	234 (37.9)	
Diabetes mellitus	208 (15.1)	102 (16.2)	21 (17.2)	85 (13.7)	0.36
Hypertension	247 (18.0)	110 (17.5)	22 (18.0)	115 (18.5)	0.90
Antiviral treatment regimen (*n* = 744)					<0.001
IFN-based therapy	418 (56.2)	—	104 (85.2)	314 (50.5)	
DAA therapy	326 (43.8)	—	18 (14.8)	308 (49.5)	

Unless otherwise indicated, data are given as number (%) of patients.

### Association between SVR and the risk of HCC occurrence

**Figure 1:**
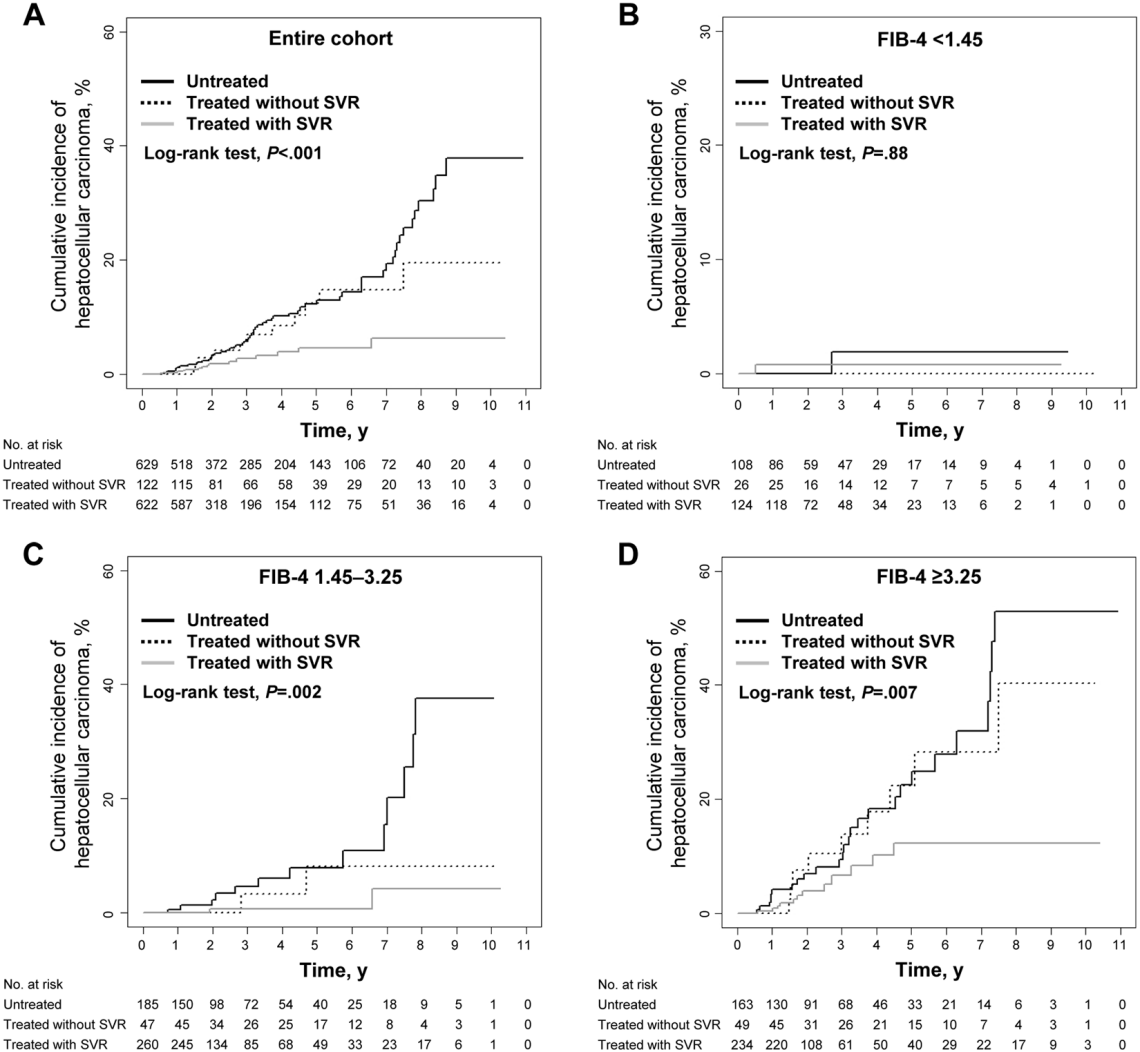
Incidence of HCC by group. (**A**) In the entire study population. (**B**) In patients with low-probability of significant fibrosis (FIB-4 < 1.45). (**C**) In patients with intermediate-probability of significant fibrosis (FIB-4 1.45–3.25). (**D**) In patients with high-probability of significant fibrosis (FIB-4 ≥ 3.25).

**Figure 2:**
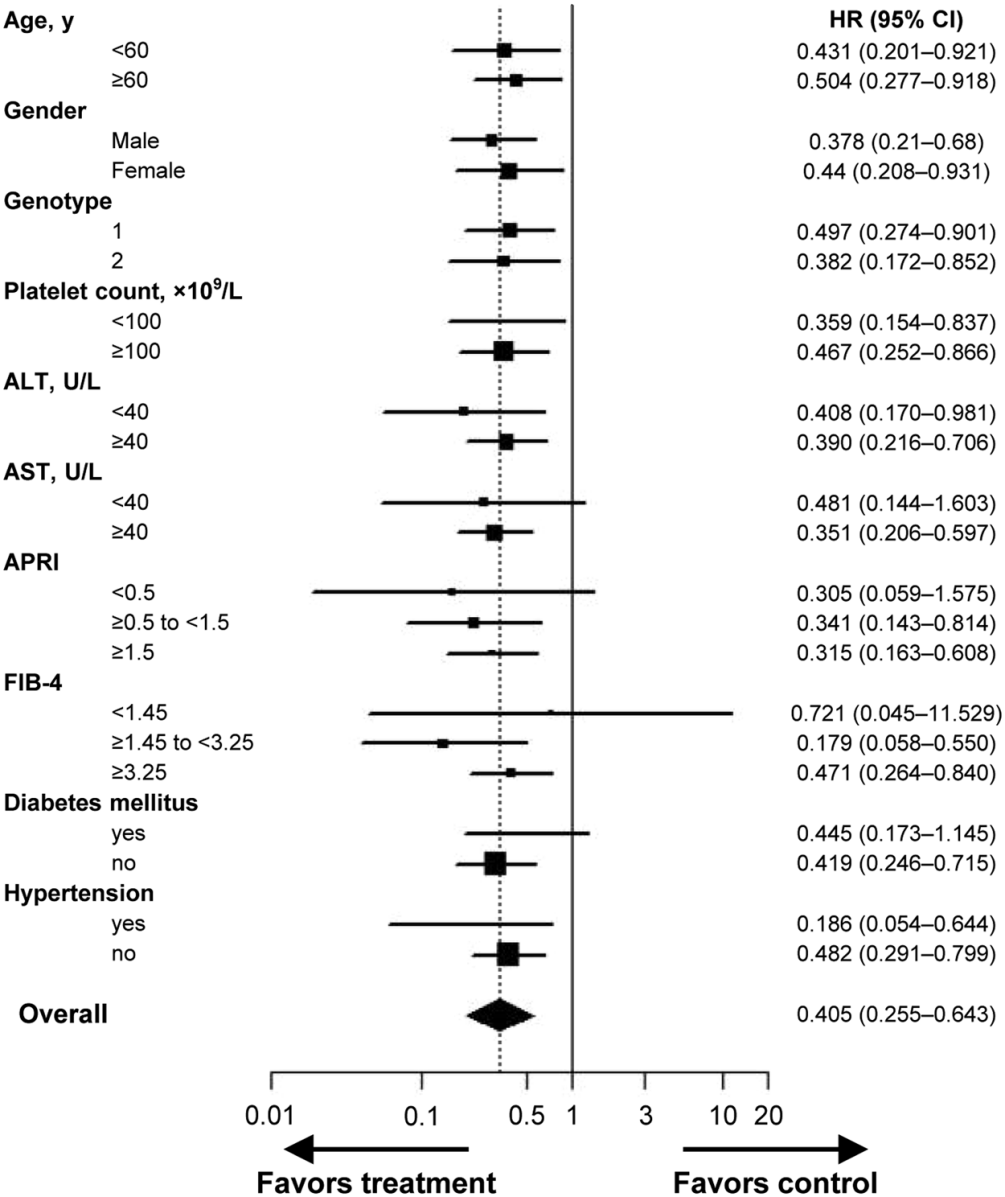
The risk of HCC in the subgroups stratified according to baseline characteristics. The graph shows the estimates of the HR for each subgroup as a *square* (which is sized proportionally to the amount of information per subgroup) and the *horizontal lines* indicate 95% CIs, those were calculated using the Cox proportional hazards model. The *vertical solid line* at the HR of unity corresponds to the line of no effect, the HR values less than unity mean a reduced risk of developing HCC by antiviral treatment. The *diamond* indicates HR with 95% CI for all study subjects and the *vertical dashed line* depicts the overall effect. Patients who received antiviral treatment were included in the treated group regardless of SVR achievement.

Eighty-five patients (6.2%) developed HCC during the observation period: 59 (9.4%) in the untreated group, 11 (9.0%) in the non-SVR group, and 15 (2.4%) in the SVR group, corresponding to 5-year cumulative incidences of 12.3%, 12.4%, and 4.6%, respectively (Table 2). There was a significant difference in the cumulative risk of HCC development between the groups (*P* < 0.001 by log-rank test) (Fig. 1A). Multivariable Cox proportional hazards regression analysis revealed that SVR achieved with treatment was associated with a significantly lower risk of HCC occurrence (*vs*. untreated; adjusted hazard ratio [HR], 0.165; 95% confidence interval [CI], 0.077–0.350; *P* < 0.001) (Table 3). Male gender and higher levels of total bilirubin, international normalised ratio, and FIB-4 were significantly associated with an increased risk of HCC development in multivariable analysis. The HRs of antiviral treatment for HCC development were estimated in the subgroups according to baseline characteristics, the association between treatment and a reduced risk of HCC was verified in most subgroups (Fig. 2). Among 85 patients who were diagnosed with HCC during the observation period, 74 patients (87.1%) were diagnosed as the Barcelona Clinic for Liver Cancer stage 0 or A, and the distributions of stage at the diagnosis of HCC were similar among the three groups (*P* = 0.09) since most of the included patients underwent regular check-up.

**Table 2:** Clinical events by group.

Outcomes		Overall	Untreated	Treated without SVR	Treated with SVR
***Overall***					
**Hepatocellular carcinoma**					
1-year	Rate, %	**0**.**7**	**1**.**3**	**0**	**0**.**3**
	Sample size	1220	518	115	587
3-year	Rate, %	**4**.**7**	**5**.**8**	**6**.**9**	**2**.**8**
	Sample size	547	285	66	196
5-year	Rate, %	**9**.**5**	**12**.**3**	**12**.**4**	**4**.**6**
	Sample size	294	143	39	110
**All-cause mortality**					
1-year	Rate, %	**0**.**1**	**0**.**2**	**0**	**0**.**2**
	Sample size	1298	567	121	610
3-year	Rate, %	**1**.**3**	**1**.**7**	**0**	**0**.**9**
	Sample size	752	398	92	262
5-year	Rate, %	**2**.**9**	**4**.**6**	**1**.**1**	**0**.**9**
	Sample size	507	258	68	181
7-year	Rate, %	**6**.**1**	**9**.**8**	**4**.**6**	**0**.**9**
	Sample size	310	170	38	102
***FIB-4* < *1***.***45***					
**Hepatocellular carcinoma**					
1-year	Rate, %	**0**.**4**	**0**	**0**	**0**.**8**
	Sample size	229	86	25	118
3-year	Rate, %	**1**.**2**	**1**.**9**	**0**	**0**.**8**
	Sample size	109	47	14	48
5-year	Rate, %	**1**.**2**	**1**.**9**	**0**	**0**.**8**
	Sample size	47	17	7	23
**All-cause mortality**					
1-year	Rate, %	**0**	**0**	**0**	**0**
	Sample size	242	95	25	112
3-year	Rate, %	**0**	**0**	**0**	**0**
	Sample size	155	64	19	72
5-year	Rate, %	**0**	**0**	**0**	**0**
	Sample size	101	42	13	46
7-year	Rate, %	**1**.**3**	**3**.**1**	**0**	**0**
	Sample size	56	26	10	20
***FIB-4* ≥ *1***.***45 to* < *3***.***25***					
**Hepatocellular carcinoma**					
1-year	Rate, %	**0**.**2**	**0**.**6**	**0**	**0**
	Sample size	440	150	45	245
3-year	Rate, %	**2**.**6**	**4**.**6**	**3**.**3**	**0**.**7**
	Sample size	183	72	26	85
5-year	Rate, %	**4**.**7**	**7**.**9**	**8**.**2**	**0**.**7**
	Sample size	106	40	17	49
**All-cause mortality**					
1-year	Rate, %	**0**.**2**	**0**	**0**	**0**.**4**
	Sample size	462	161	47	254
3-year	Rate, %	**1**.**9**	**2**.**5**	**0**	**1**.**8**
	Sample size	244	103	33	108
5-year	Rate, %	**3**.**3**	**4**.**7**	**3**.**0**	**1**.**8**
	Sample size	169	66	25	78
7-year	Rate, %	**5**.**6**	**8**.**6**	**8**.**4**	**1**.**8**
	Sample size	100	42	14	44
**FIB-4 ≥ 3**.**25**					
**Hepatocellular carcinoma**					
1-year	Rate, %	**1**.**7**	**4**.**2**	**0**	**0**.**4**
	Sample size	295	130	43	195
3-year	Rate, %	**8**.**6**	**9**.**4**	**13**.**9**	**6**.**7**
	Sample size	155	68	26	57
5-year	Rate, %	**18**.**0**	**22**.**6**	**22**.**3**	**12**.**3**
	Sample size	88	33	15	40
**All-cause mortality**					
1-year	Rate, %	**0**	**0**	**0**	**0**
	Sample size	425	146	49	230
3-year	Rate, %	**1**.**1**	**1**.**8**	**0**	**0**.**5**
	Sample size	219	100	40	79
5-year	Rate, %	**4**.**6**	**9**.**8**	**0**	**0**.**5**
	Sample size	146	61	30	55
7-year	**Rate**, **%**	**8**.**7**	**17**.**7**	**3**.**4**	**0**.**5**
	Sample size	86	35	14	37

**Table 3:** Univariable and multivariable analysis of the clinical factors predictive of HCC development and all-cause mortality.

	HCC development				All-cause mortality			
	Univariable analysis		Multivariable analysis		Univariable analysis		Multivariable analysis	
	Hazard ratio (95% CI)	P value	Adjusted hazard ratio (95% CI)	P value	Hazard ratio (95% CI)	P value	Adjusted hazard ratio (95% CI)	P value
Age, y	1.058 (1.035–1.082)	<0.001			1.064 (1.034–1.095)	<0.001		
Male	2.282 (1.471–3.541)	<0.001	2.261 (1.268–4.032)	0.009	1.898 (1.064–3.387)	0.03		
Genotype		0.06				0.09		
1	1 [Reference]				1 [Reference]			
2	0.572 (0.359–0.913)	0.02			0.687 (0.371–1.274)	0.23		
Other	0.469 (0.065–3.396)	0.45			2.567 (0.773–8.525)	0.12		
Platelet count, ×10^9^/L	0.983 (0.979–0.988)	<0.001			0.99 (0.984–0.996)	<0.001		
Albumin, g/dL	0.157 (0.093–0.263)	<0.001			0.109 (0.054–0.219)	<0.001	0.144 (0.065–0.319)	<0.001
Total bilirubin, mg/dL	2.766 (1.733–4.414)	<0.001	2.051 (1.122–3.75)	0.03	2.758 (1.432–5.311)	0.002	3.483 (1.657–7.318)	<0.001
ALT, IU/L	0.997 (0.994–1.001)	0.12			0.996 (0.991–1.001)	0.11		
AST, IU/L	1.001 (0.999–1.004)	0.24			1 (0.994–1.005)	0.95		
GGT, IU/L	1.003 (1.001–1.005)	<0.001	1.002 (1–1.005)	0.047	1.002 (0.999–1.005)	0.15		
INR	5.937 (2.313–15.235)	<0.001	6.331 (1.409–28.454)	0.01	12.666 (4.633–34.628)	<0.001		
AFP, ng/mL	1 (0.999–1.001)	0.99			1.000 (0.999–1.001)	0.95		
HCV RNA, log^10^ IU/ml	0.892 (0.707–1.126)	0.34			0.906 (0.663–1.239)	0.54		
APRI		<0.001				0.21		
<0.5	1 [Reference]				1 [Reference]			
≥0.5 to <1.5	1.91 (0.816–4.473)	0.14			1.569 (0.608–4.047)	0.35		
≥1.5	5.133 (2.286–11.525)	<0.001			2.278 (0.882–5.883)	0.09		
FIB-4		<0.001		0.002		0.02		
<1.45	1 [Reference]		1 [Reference]		1 [Reference]			
≥1.45 to <3.25	4.377 (1.011–18.951)	0.05	2.403 (0.54–10.692)	0.25	8.155 (1.072–62.021)	0.04		
≥3.25	14.057 (3.413–57.896)	<0.001	5.814 (1.368–24.709)	0.02	13.861 (1.864–103.07)	0.01		
Diabetes mellitus								
No	1 [Reference]				1 [Reference]			
Yes	2.008 (1.244–3.241)	0.004			1.79 (0.931–3.44)	0.08		
Hypertension								
No	1 [Reference]				1 [Reference]			
Yes	1.256 (0.746–2.115)	0.39			1.21 (0.585–2.499)	0.61		
Treatment		<0.001		<0.001		<0.001		<0.001
Untreated	1 [Reference]		1 [Reference]		1 [Reference]		1 [Reference]	
Treated wihout SVR	0.733 (0.385–1.397)	0.35	0.658 (0.313–1.383)	0.27	0.314 (0.097–1.016)	0.05	0.099 (0.013–0.731)	0.02
Treated wih SVR	0.304 (0.172–0.537)	<0.001	0.165 (0.077–0.35)	<0.001	0.173 (0.068–0.44)	<0.001	0.146 (0.05–0.424)	<0.001

*P* values were determined using Cox proportional hazards regression models. *P* < 0.05 indicated a significant difference.

Variables in the multivariable analysis were selected using stepwise regression with the forward selection method.

When patients were categorised into the three subgroups according to the probabilities of significant hepatic fibrosis (Tables S1), 2 of 258 patients (0.8%) with low-probability of significant fibrosis (FIB-4 < 1.45), 16 of 492 patients (3.3%) with intermediate-probability (FIB-4 1.45–3.25), and 42 of 446 patients (9.4%) with high-probability (FIB-4 ≥ 3.25) developed HCC during follow-up, which corresponded to the 5-year cumulative risks of HCC of 1.2%, 4.7%, and 18.0%, respectively (Table 2). The risk of developing HCC rose with increasing FIB-4 scores at baseline (*P* < 0.001) (Fig. S1A). The cumulative incidences of HCC did not differ significantly between the groups among the subgroup of low-probability of significant fibrosis (*vs*. untreated; adjusted HR, 0.884; 95% CI, 0.044–17.637; *P* = 0.94) (Table 4 and Fig. 1B), whereas the cumulative incidences of HCC were significantly lower in the SVR group among the subgroups of intermediate-probability (*vs*. untreated; adjusted HR, 0.171; 95% CI, 0.051–0.578; *P* = 0.004) and high-probability (*vs*. untreated; adjusted HR, 0.243; 95% CI, 0.107–0.551; *P* < 0.001) (Table 4 and Fig. 1C,D).

**Table 4:** Association between SVR and the risk of HCC among subgroups according to the probabilities of significant fibrosis.

	HCC development		All-cause mortality	
	Adjusted hazard ratio (95% CI)	*P* value	Adjusted hazard ratio (95% CI)	*P* value
***FIB-4 < 1***.***45***				
Untreated	1 [Reference]		1 [Reference]	
Treated without SVR	1.307 (0.019–90.273)	0.90	0.970 (0.006–152.889)	0.99
Treated with SVR	0.884 (0.044–17.637)	0.94	0.296 (0.002–46.728)	0.64
***FIB-4 ≥ 1***.***45 to < 3***.***25***				
Untreated	1 [Reference]		1 [Reference]	
Treated without SVR	0.124 (0.016–0.939)	0.04	0.817 (0.172–3.89)	0.8
Treated with SVR	0.171 (0.051–0.578)	0.004	0.574 (0.165–1.995)	0.38
***FIB-4 ≥ 3***.***25***				
Untreated	1 [Reference]		1 [Reference]	
Treated without SVR	0.928 (0.43–2.002)	0.85	0.104 (0.014–0.783)	0.03
Treated with SVR	0.243 (0.107–0.551)	<0.001	0.057 (0.007–0.431)	0.006

*P* values were determined using multivariable Cox proportional hazards regression models.

After balancing baseline characteristics by means of IPW, baseline characteristics became well balanced between the groups (Tables S2–S8). The association of SVR with a reduced risk of HCC was confirmed in the entire cohort (*vs*. untreated; adjusted HR, 0.226; 95% CI, 0.099–0.521; *P* < 0.001) and in the subgroups of intermediate-probability (*vs*. untreated; adjusted HR, 0.033; 95% CI, 0.002–0.706; *P* = 0.03) and high-probability (*vs*. untreated; adjusted HR, 0.305; 95% CI, 0.126–0.739; *P* = 0.009) of significant fibrosis (Tables S9–S10 and Fig. S2).

### Association between SVR and the risk of all-cause mortality

**Figure 3:**
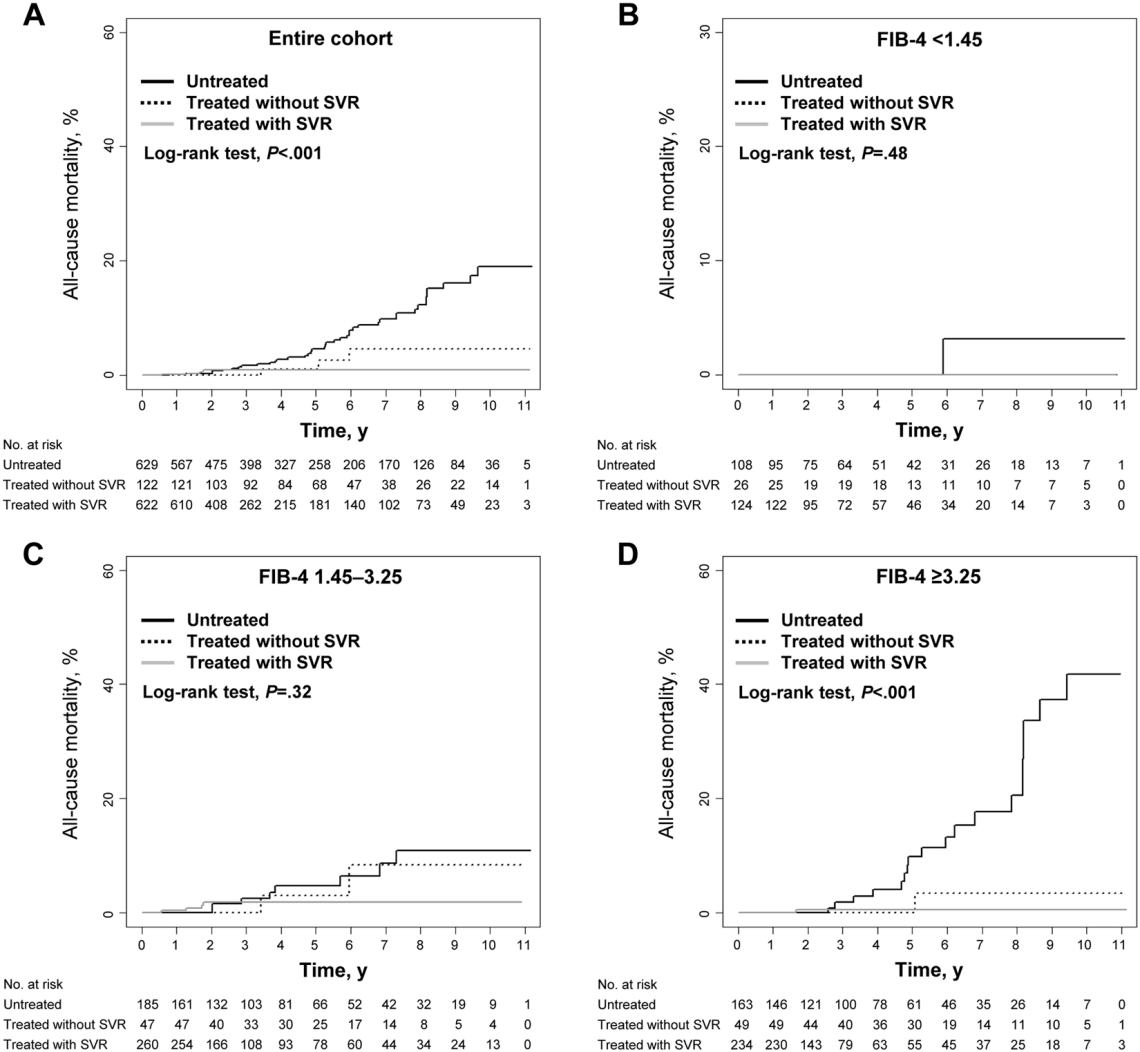
Incidence of all-cause mortality by group. (**A**) In the entire study population. (**B**) In patients with low-probability of significant fibrosis (FIB-4 < 1.45). (**C**) In patients with intermediate-probability of significant fibrosis (FIB-4 1.45–3.25). (**D**) In patients with high-probability of significant fibrosis (FIB-4 ≥ 3.25).

**Figure 4:**
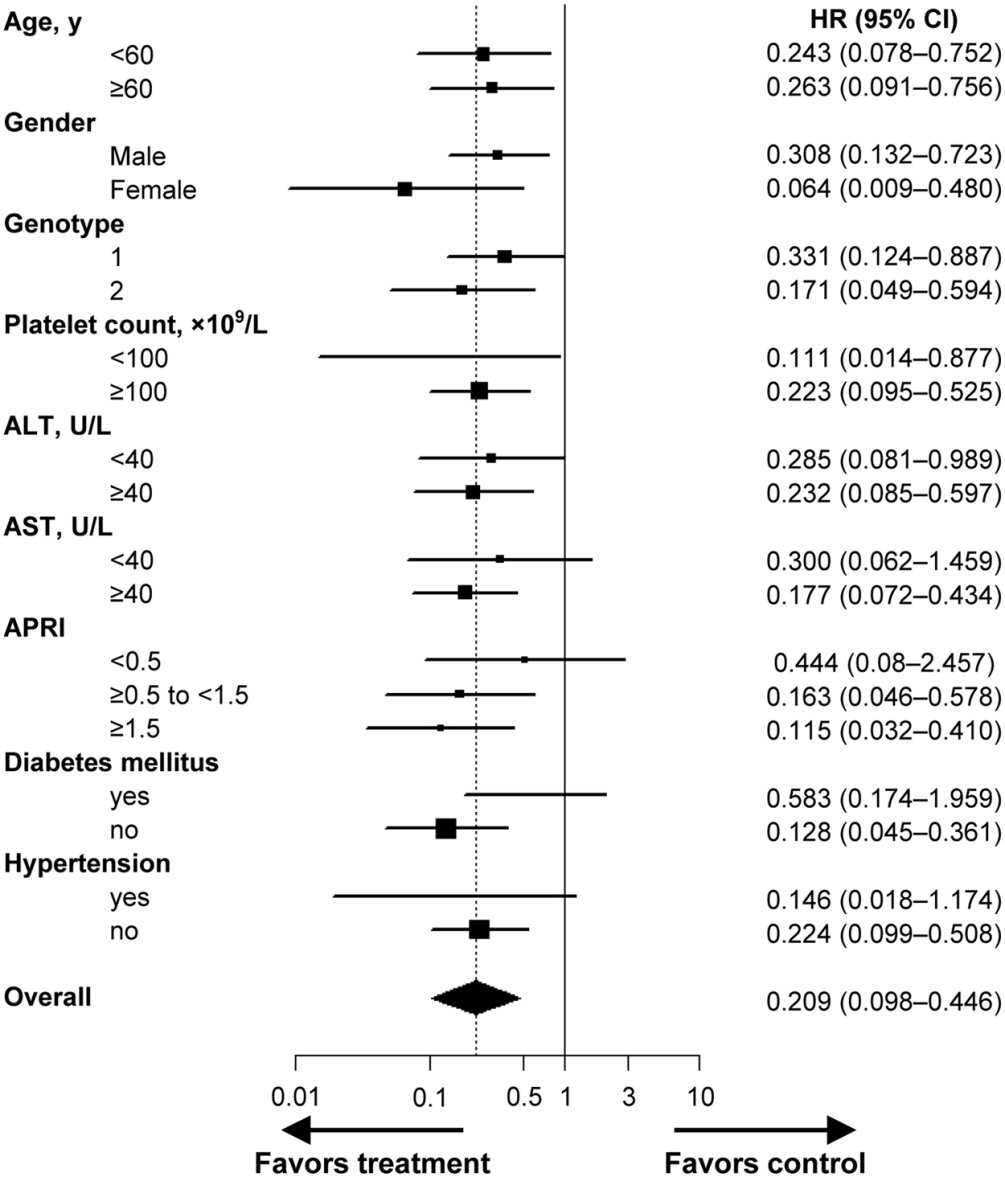
The risk of all-cause mortality in the subgroups stratified according to baseline characteristics. The graph shows the estimates of the HR for each subgroup as a *square* (which is sized proportionally to the amount of information per subgroup) and the *horizontal lines* indicate 95% CIs, those were calculated using the Cox proportional hazards model. The *vertical solid line* at the HR of unity corresponds to the line of no effect, the HR values less than unity mean a reduced risk of all-cause mortality by antiviral treatment. The *diamond* indicates HR with 95% CI for all study subjects and the *vertical dashed line* depicts the overall effect. Patients who received antiviral treatment were included in the treated group regardless of SVR achievement.

Forty-eight deaths occurred in the entire cohort during the follow-up: 40 (6.4%) in the untreated group, 3 (2.5%) in the non-SVR group, and 5 (0.8%) in the SVR group. Among 28 patients whose causes of death were identified, 26 died of liver-related complications, including HCC, and the two remaining deaths were not liver-related. No significant difference was observed among the three groups (P = 0.11 by log-rank test) when overall survival was analysed after the HCC diagnosis. Causes of non-liver-related death were extrahepatic malignancy (*n* = 1) and advanced pulmonary disease (*n* = 1). The 5-year cumulative all-cause mortality rate was 2.9% overall, and differed significantly between the groups: 4.6%, 1.1%, and 0.9% in the untreated, the non-SVR, and the SVR group, respectively (*P* < 0.001 by log-rank test) (Table 2 and Fig. 3A). Table 3 shows independent predictive factors associated with all-cause mortality in the entire study population, in which SVR achieved with antiviral treatment was a predictive factor for a reduced risk of overall death selected by multivariable analysis (*vs*. untreated; adjusted HR, 0.146; 95% CI, 0.050–0.424; *P* < 0.001). Subgroup analyses demonstrated that the association between treatment and improved mortality remained statistically significant in most subgroups (Fig. 4).

One patient (0.4%), 14 patients (2.8%), and 21 patients (4.7%) among patients with low-, intermediate-, and high-probability of significant fibrosis died during the observation period, respectively, with the corresponding cumulative 5-year mortality rates of 0%, 3.3%, and 4.6%, respectively (Table 2). The cumulative mortality rates varied significantly by fibrosis stage assessed using FIB-4 scores (*P* = 0.003) (Fig. S1B). While the cumulative incidence rates of overall death were not significantly different between the groups among the subgroups of low-probability (*vs*. untreated; adjusted HR, 0.296; 95% CI, 0.002–46.728; *P* = 0.64) and intermediate-probability (*vs*. untreated; adjusted HR, 0.574; 95% CI, 0.165–1.995; *P* = 0.38) of significant fibrosis (Table 4 and Fig. 3B,C), a statistically significant difference was identified among the subgroup of high-probability (*vs*. untreated; adjusted HR, 0.057; 95% CI, 0.007–0.431; *P* = 0.006) (Table 4 and Fig. 3D).

After IPW, the patients in the SVR group were at significantly lower risk of mortality in the overall population (*vs*. untreated; adjusted HR, 0.171; 95% CI, 0.051–0.577; *P* = 0.004) and in the subgroup of high-probability of significant fibrosis (*vs*. untreated; adjusted HR, 0.033; 95% CI, 0.002–0.683; *P* = 0.03) (Tables S9–S10 and Fig. S3).

### Association of type of antiviral treatment regimen with the risk of HCC and all-cause mortality

Among 622 patients who achieved SVR with antiviral treatment, 314 patients received IFN-based therapy and 308 received DAA therapy. As expected, patients treated with DAA therapy were older and showed lower platelet count and serum albumin level, and higher FIB-4 scores compared with patients treated with IFN-based therapy (Table S11). The difference in the cumulative risk of HCC development between the groups was significant (*P* = 0.006 by log-rank test) (Fig. S4A), while the difference in the cumulative risk of overall death was not (*P* = 0.45 by log-rank test) (Fig. S4B). However, type of antiviral agent that induced SVR was not associated with HCC risk (*vs*. IFN-based therapy; adjusted HR, 4.289; 95% CI, 0.648–28.408; *P* = 0.13) and the risk of all-cause mortality (*vs*. IFN-based therapy; adjusted HR, 1.348; 95% CI, 0.208–8.735; *P* = 0.75) in the multivariable Cox model. No significant association between antiviral agent and the risk of HCC and all-cause mortality was found even after adjusting for differences in baseline characteristics using IPW (Table S12 and Fig. S4C,D).

## Discussion

In our study, SVR achieved with antiviral treatment including IFN-based therapy and DAAs was associated with a reduced risk of developing HCC. The risk of HCC development was significantly lower by 84% in patients who achieved SVR with antiviral treatment compared with untreated patients. The association between SVR and the risk of HCC was evident among patients with intermediate- or high-probability of significant hepatic fibrosis (i.e., FIB-4 ≥ 1.45) at baseline. In addition, we demonstrated an association between SVR achieved with treatment and prolonged overall survival, especially among patients with high-probability of significant hepatic fibrosis (i.e., FIB-4 ≥ 3.25) at baseline. DAA-induced SVR was not associated with increased risk of HCC and overall death compared with SVR achieved with IFN-based therapy.

We identified the beneficial impact of SVR on the risk of HCC development according to stratified probabilities of significant fibrosis based on a noninvasive marker, the FIB-4 score. We observed increasing risk of HCC in proportion to baseline FIB-4 scores. Additionally, SVR was associated with a relatively less reduction of HCC risk among patients with high-probability of significant hepatic fibrosis (adjusted HR, 0.243; 95% CI, 0.107–0.551) than among patients with intermediate-probability of significant hepatic fibrosis (adjusted HR, 0.171; 95% CI, 0.051–0.578), suggesting the beneficial effect of earlier treatment initiation before progression to advanced fibrosis or cirrhosis. A previous study using the US Veterans Administration database showed early treatment before FIB-4 reaching 3.25 was more beneficial than deferring treatment after FIB-4 exceeding 3.25 in terms of mortality and liver-related morbidity^21^. Although effect of SVR on mortality and liver-related morbidity by fibrosis stage cannot be assessed clearly because data on SVR achievement with treatment are not available in the previous report, the study results are supportive of our present study.

Assessment of liver disease severity prior to antiviral therapy is mandatory for the prediction of treatment response and long-term outcomes^2,4^. FIB-4, an accurate, inexpensive, and noninvasive marker of hepatic fibrosis in HCV-infected patients, has the strong advantage of availability to all clinical practitioners, even in developing countries where medical resources are limited^22^. Accordingly, FIB-4 is recommended as one of the most useful noninvasive tests for assessing the severity of liver disease by the current international guideline^2^. A FIB-4 score < 1.45 showed a negative predictive value for the exclusion of severe fibrosis (≥F3) of 94.7%, while a score ≥ 3.25 had a positive predictive value of 82.1% to confirm severe fibrosis^22^. In our study, SVR was associated with reduced risk of all-cause mortality and HCC among patients with high-probability of significant fibrosis at baseline, these findings were consistent with the previous reports^15,16^. A recent study conducted by Yu *et al*. showed that non-SVR was an independent predictor of HCC development in patients with METAVIR fibrosis grade F2–F3 or F4 but not in those with F0–F1^23^. Although that previous study included only patients receiving IFN-based therapy, the results are generally consistent with our present study. Moreover, we additionally demonstrated that patients with high-probability of significant fibrosis were not at low risk of developing HCC, even if they receive antiviral treatment and achieve SVR. The 5-year cumulative incidence rate of HCC was 12.3% in the SVR group among patients with high-probability of significant fibrosis at baseline, as distinguished from extremely low rate in the SVR group among patients with low- or intermediate-probability (i.e., FIB-4 < 3.25). Consequently, prompt treatment initiation is absolutely indicated for patients with high-probability of significant fibrosis and these patients need to undergo ongoing surveillance for HCC even after achieving SVR, given that their estimated HCC risk exceeds the thresholds (0.8–1.5% per year) for cost-effectiveness of HCC surveillance^2–4,24,25^. Kanwal *et al*. recently reported that annual incidences of HCC ranged from 1.0% to 2.2% among virologically cured patients with cirrhosis diagnosis^11^. Moreover, HCC risk was not low (2.16% per year) in patients with a high baseline FIB-4 (>3.25) in that study. In contrast, it was determined that successful HCV eradication diminished and nearly eliminated the risk of HCC in patients with low- or intermediate-probability of significant fibrosis in our present study, thus further studies to investigate whether these patients should remain under regular surveillance for HCC are warranted.

In our current study, we demonstrated that patients who achieved SVR with antiviral treatment faced reduced risk of HCC (84%) and all-cause mortality (85%) than untreated patients. In the secondary analysis involving patients in whom SVR was achieved with antiviral treatment, it was found that DAA-induced SVR was not associated with higher risk of HCC and overall death compared with SVR induced by IFN-based therapy. These findings are consistent with a recent study conducted by Ioannou *et al*. using the US Veterans Administration cohort^12^. Although that previous study only included treated patients and compared the risk of HCC between the SVR group and the non-SVR group, SVR was associated with a 61% reduction in HCC risk compared with treatment failure. Furthermore, the risk reduction of HCC associated with SVR was similar regardless of treatment regimen (DAA-only, DAA + IFN, or IFN-only), leading to the conclusion that there was no evidence supporting the hepatocarcinogenic effect of DAA therapy. However, because the follow-up period of patients treated with DAAs was relatively short compared to that of patients treated with IFN-based therapy, long-term comparative studies are warranted.

An untreated control group was included in our present study to clearly assess the effect of antiviral treatment on the risk of HCC and mortality. However, substantial bias inherent in this approach should be concerned. The untreated patients might be less likely to be diagnosed with HCC than the treated patients because of lower compliance to HCC surveillance. Considering this possible ascertainment bias, the actual cumulative HCC incidence curves are presumed to further diverge than those of the current study. Moreover, we took into account the inevitable selection bias resulting from the nature of retrospective observational study design, and differences in some baseline characteristics between the groups were observed indeed. Thus, we performed rigorous adjustment for confounding factors by means of employment of IPW to reduce the treatment selection bias.

Our study has several limitations. First, since this was a retrospective study, we could not obtain data on metabolic features of the study subjects, such as body mass index and the presence of dyslipidaemia. However, given that the presence of diabetes mellitus or hypertension was not significantly associated with HCC development or overall death in our study, it is unlikely that other metabolic features had a considerable impact on the study results. Second, owing to missing values of certain baseline characteristics for calculation of propensity scores in a considerable number of patients, the sample size for IPW analysis became relatively small. Nevertheless, the analyses before and after IPW were generally consistent. Third, the follow-up period of patients treated with DAAs was not long enough to assess long-term effects of DAA-induced SVR. While the SVR effect on the risk of HCC and mortality was not found among patients with low-probability of significant hepatic fibrosis (FIB-4 < 1.45) in our present study, it may become evident with accumulation of clinical events over time^26^.

In summary, reduced risk of developing HCC and all-cause mortality was observed in patients with chronic HCV infection achieving viral eradication with antiviral treatment including IFN-based therapy and DAAs, and the beneficial effect of SVR on the risk of HCC was verified in patients with intermediate- or high-probability of significant fibrosis (FIB-4 ≥ 1.45). However, since SVR was not directly linked to complete prevention of HCC development in patients with high-probability of significant hepatic fibrosis, regular HCC surveillance may be necessary in these patients. In addition, we found that patients achieving SVR with DAA therapy were not at higher risk of HCC and overall death compared with patients achieving SVR with IFN-based therapy.

## Patients and Methods

### Study population

The study population was obtained from inpatient and outpatient database files between January 1, 2006 and January 31, 2017 at Seoul National University Hospital (Seoul, Korea) and consisted of a cohort of 1,899 consecutive CHC patients positive for both HCV antibody (anti-HCV) and serum HCV RNA (Fig. S5). Exclusion criteria were (1) history of malignant disease including HCC; (2) prior liver transplantation; (3) co-infection with hepatitis B virus or human immunodeficiency virus; (4) history of chronic kidney disease or heart failure; (5) hepatic decompensation; (6) follow-up duration of less than 6 months; and (7) diagnosis of HCC within 6 months of follow-up. Consequently, a total of 1,373 patients were included in the final analyses.

Patients in the treated group were treated with IFN-based regimens including standard and pegylated IFN with or without ribavirin (but without any of DAAs) or DAAs with or without ribavirin. SVR was defined as undetectable serum HCV RNA level by the Abbott Real-Time PCR HCV assay (Abbott Molecular Inc., IL, USA) with a lower limit of detection of 12 IU/mL at least 12 weeks post-treatment^27^. Patients in the untreated group were not treated due to unwillingness to receive antiviral treatment with concern about high cost and possible drug-drug interactions of DAA-based treatment or low SVR rates and side effects of IFN-based treatment.

Patients were grouped into three groups: the untreated group; the non-SVR group; and the SVR group. FIB-4 scores, a simple noninvasive fibrosis scoring system, were calculated using the following formula: age (years) × aspartate aminotransferase (AST) (U/L)/[platelets (10^9^/L) × [alanine aminotransferase (ALT) (U/L)]^1/2^], and patients were categorised into the following subgroups according to FIB-4 scores at baseline: < 1.45, low-probability of significant fibrosis, *n* = 258; 1.45–3.25, intermediate-probability, *n* = 492; and ≥ 3.25, high-probability, *n* = 446^22^.

The study conformed to the ethical guidelines of the World Medical Association Declaration of Helsinki and was approved by the Institutional Review Board of Seoul National University Hospital. The requirement for written informed consent was waived, because clinical data were analysed anonymously.

### Clinical outcome measures

The primary outcome was the development of HCC during the observation period. The secondary outcome was all-cause mortality. The index date was defined as the first date that a patient was found to have a positive anti-HCV test for the untreated group and the date of treatment initiation for the treated group regardless of SVR achievement. Most study subjects underwent surveillance for HCC with abdominal imaging and/or alpha-fetoprotein. Diagnosis of HCC was established based on guidelines of the American Association for the Study of Liver Diseases^24,28^. All image scans were reviewed by two radiologists (L.J.M. and L.D.H.) with >10 years of experience who were unaware of the clinical information for the study patients. An additional independent experienced radiologist reviewed radiological images in cases of discordance. If diagnosis was not established radiologically, we performed liver biopsy for histological diagnosis of HCC.

### Statistical analysis

For the primary analysis, the baseline demographic and clinical characteristics were compared among the three groups of study subjects. For group-wise comparisons, one-way analysis of variance or the Kruskal-Wallis test was used for continuous variables, and either the χ^2^ test or the Fisher’s exact test was performed for categorical variables. The Mantel-Byar method was applied to correct immortal time bias^29,30^. If the date of anti-HCV testing and the date of treatment initiation were more than 6 months apart in treated patients, the time between the two time points was regarded as “untreated” and “treated” thereafter. In the time-to-event analysis, if patients in the untreated group started antiviral treatment, patients were censored at the date of initiation of antiviral treatment. If patients who had failed to achieve SVR with IFN-based therapy (the non-SVR group) were retreated with DAA therapy, patients were censored at the date of initiation of DAA therapy. Patients lost to follow-up before the diagnosis of HCC were censored at the date of the last surveillance for HCC in the time-to-HCC development analysis, whereas patients were not censored except in case of aforementioned patient censoring in the time-to-all-cause mortality analysis. Times to events and cumulative incidences were calculated using the Kaplan–Meier method and compared by the log-rank test. The treatment effect on occurrence of HCC and all-cause mortality was assessed using the Cox proportional hazards regression model. Subgroup analyses were conducted to further investigate the impact of SVR.

Inverse probability weighting (IPW) based on propensity score that estimated the probability to be treated and achieve SVR was applied to correct baseline differences among the three groups^31,32^. A propensity score for each patient was calculated using a logistic regression model including the baseline demographic and clinical characteristics as described previously^33^. The baseline characteristics between the groups were more balanced after IPW, and we fitted weighted Cox models thereafter.

For the secondary analysis, the patients who achieved SVR were divided into two groups according to type of antiviral regimen (IFN-based therapy or DAA therapy). The baseline characteristics were compared between the two groups and the effect on the risk of HCC and mortality was evaluated. We re-calculated propensity score of each patient reflecting the probability to be treated with DAAs and performed IPW, and then, survival analyses were repeated.

All statistical tests were conducted as two-sided tests, and a *P* value less than 0.05 was considered statistically significant. R language version 3.3.0 (R Foundation for Statistical Computing, Vienna, Austria) and SAS software version 9.4 (SAS Inc., Cary, NC, USA) were used for all statistical analyses.

## Electronic supplementary material

Supplementary Material

## References

[1] Lavanchy D (2011). Evolving epidemiology of hepatitis C virus. Clin Microbiol Infect.

[2] World Health Organization. Guidelines for the screening, care and treatment of persons with chronic hepatitis C infection. http://www.who.int/hepatitis/publications/hepatitis-c-guidelines-2016/en/. Last accessed 7/20/2018.

[3] AASLD-IDSA. Recommendations for testing, managing, and treating hepatitis C. http://www.hcvguidelines.org/full-report-view. Last accessed 4/24/2018.

[4] European Association for the Study of the Liver. EASL recommendations on treatment of hepatitis C 2016. *J*. *Hepatol*. **66**, 153–194, 10.1016/j.jhep.2016.09.001 (2017). 10.1016/j.jhep.2022.10.006 36464532

[5] Morgan RL (2013). Eradication of hepatitis C virus infection and the development of hepatocellular carcinoma: a meta-analysis of observational studies. Ann Intern Med.

[6] Reig M (2016). Unexpected high rate of early tumor recurrence in patients with HCV-related HCC undergoing interferon-free therapy. J Hepatol.

[7] Conti F (2016). Early occurrence and recurrence of hepatocellular carcinoma in HCV-related cirrhosis treated with direct-acting antivirals. J Hepatol.

[8] ANRS collaborative study group on hepatocellular carcinoma (ANRS CO22 HEPATHER, CO12 CirVir and CO23 CUPILT cohorts). Lack of evidence of an effect of direct-acting antivirals on the recurrence of hepatocellular carcinoma: Data from three ANRS cohorts. *J Hepatol* **65**, 734–740, 10.1016/j.jhep.2016.05.045 (2016). 10.1016/j.jhep.2016.05.045 27288051

[9] Cheung MC (2016). Outcomes after successful direct-acting antiviral therapy for patients with chronic hepatitis C and decompensated cirrhosis. J Hepatol.

[10] Reig M (2017). Liver cancer emergence associated with antiviral treatment: an immune surveillance failure?. Semin Liver Dis.

[11] Kanwal F (2017). Risk of hepatocellular cancer in HCV patients treated With direct-acting antiviral agents. Gastroenterology.

[12] Ioannou George N., Green Pamela K., Berry Kristin (2018). HCV eradication induced by direct-acting antiviral agents reduces the risk of hepatocellular carcinoma. Journal of Hepatology.

[13] Bruden DJ (2017). Risk of end stage liver disease, hepatocellular carcinoma and liver-related death by fibrosis stage in the hepatitis C Alaska Cohort. Hepatology.

[14] Xu F (2016). All-cause mortality and progression risks to hepatic decompensation and hepatocellular carcinoma in patients infected with hepatitis C virus. Clin Infect Dis.

[15] van der Meer AJ (2012). Association between sustained virological response and all-cause mortality among patients with chronic hepatitis C and advanced hepatic fibrosis. JAMA.

[16] Nahon P (2017). Eradication of hepatitis C virus infection in patients with cirrhosis reduces risk of liver and non-Liver complications. Gastroenterology.

[17] Janjua NZ (2017). Long-term effect of sustained virological response on hepatocellular carcinoma in patients with hepatitis C in Canada. J Hepatol.

[18] Backus LI (2011). A sustained virologic response reduces risk of all-cause mortality in patients with hepatitis C. Clin Gastroenterol Hepatol.

[19] Dienstag JL (2011). A prospective study of the rate of progression in compensated, histologically advanced chronic hepatitis C. Hepatology.

[20] Mira JA (2013). Benefits from sustained virologic response to pegylated interferon plus ribavirin in HIV/hepatitis C virus-coinfected patients with compensated cirrhosis. Clin Infect Dis.

[21] McCombs, J., Tonnu-MiHara, I., Matsuda, T., McGinnis, J. & Fox, S. Can hepatitis c treatment be safely delayed? Evidence from the veterans administration healthcare system. 50th Annual Meeting of the European Association for the Study of the Liver (EASL). *J Hepatol* **62**, S191 (2015).

[22] Vallet-Pichard A (2007). FIB-4: an inexpensive and accurate marker of fibrosis in HCV infection. comparison with liver biopsy and fibrotest. Hepatology.

[23] Yu ML (2017). Time-degenerative factors and the risk of hepatocellular carcinoma after antiviral therapy among hepatitis C virus patients: A model for prioritization of treatment. Clin Cancer Res.

[24] Bruix J, Sherman M (2011). Management of hepatocellular carcinoma: an update. Hepatology.

[25] Cucchetti A, Cescon M, Erroi V, Pinna AD (2013). Cost-effectiveness of liver cancer screening. Best Pract Res Clin Gastroenterol.

[26] Backus LI, Belperio PS, Shahoumian TA, Mole LA (2018). Direct-acting antiviral sustained virologic response: Impact on mortality in patients without advanced liver disease. Hepatology.

[27] Korean Association for the Study of the Liver. KASL clinical practice guidelines: management of hepatitis C. *Clin Mol Hepatol* **22**, 76–139, 10.3350/cmh.2016.22.1.76 (2016). 10.3350/cmh.2016.22.1.76 PMC4825161 27044763

[28] Bruix J, Sherman M (2005). Management of hepatocellular carcinoma. Hepatology.

[29] Mantel N, Byar DP (1974). Evaluation of response-time data involving transient states: An illustration using heart-transplant data. Journal of the American Statistical Association.

[30] Suissa S (2008). Immortal time bias in pharmaco-epidemiology. Am J Epidemiol.

[31] Rosenbaum PR, Rubin DB (1983). The central role of the propensity score in observational studies for causal effects. Biometrika.

[32] Curtis LH, Hammill BG, Eisenstein EL, Kramer JM, Anstrom KJ (2007). Using inverse probability-weighted estimators in comparative effectiveness analyses with observational databases. Med Care.

[33] Lee YB (2014). Efficacy of entecavir-tenofovir combination therapy for chronic hepatitis B patients with multidrug-resistant strains. Antimicrob Agents Chemother.

