# Publisher Correction: Differential Effect of HCV Eradication and Fibrosis Grade on Hepatocellular Carcinoma and All-cause Mortality

**DOI:** 10.1038/s41598-019-48367-y

**Published:** 2019-08-16

**Authors:** Yun Bin Lee, Joon Yeul Nam, Jeong-Hoon Lee, Young Chang, Hyeki Cho, Young Youn Cho, Eun Ju Cho, Su Jong Yu, Hwi Young Kim, Dong Ho Lee, Jeong Min Lee, Seong Gyu Hwang, Yoon Jun Kim, Jung-Hwan Yoon

**Affiliations:** 1 Department of Internal Medicine and Liver Research Institute, Seoul National University College of Medicine, Seoul, Korea; 2 Department of Internal Medicine, CHA Bundang Medical Center, CHA University, Seongnam, Korea; 3 Department of Internal Medicine and Liver Center, Ewha Womans University School of Medicine, Seoul, Korea; 4 Department of Radiology, Seoul National University College of Medicine, Seoul, Korea

Correction to: *Scientific Reports* 10.1038/s41598-018-31839-y, published online 12 September 2018

In the original version of this Article, the author Yun Bin Lee was incorrectly indexed. This error has now been corrected.

