# Supplementary material for: Differential Effect of HCV Eradication and Fibrosis Grade on Hepatocellular Carcinoma and All-cause Mortality
Source: Sci Rep. 2018 Sep 12;8:13651. doi: 10.1038/s41598-018-31839-y (PMC6135856; doi:10.1038/s41598-018-31839-y)

Online-only supplements

Differential effect of HCV eradication and fibrosis grade on hepatocellular carcinoma and all-cause mortality

Yun Bin Lee[1,2,*], Joon Yeul Nam[1,*], Jeong-Hoon Lee[1], Young Chang[1], Hyeki Cho[1], Young Youn Cho[1], Eun Ju Cho[1], Su Jong Yu[1], Hwi Young Kim[3], Dong Ho Lee[4], Jeong Min Lee[4], Seong Gyu Hwang[2], Yoon Jun Kim[1], Jung-Hwan Yoon[1]

[1]*Department of Internal Medicine and Liver Research Institute, Seoul National University College of Medicine, Seoul, Korea*

[2]*Department of Internal Medicine, CHA Bundang Medical Center, CHA University, Seongnam, Korea*

[3]*Department of Internal Medicine and Liver Center, Ewha Womans University School of Medicine, Seoul, Korea*

[4]*Department of Radiology, Seoul National University College of Medicine, Seoul, Korea*

* These authors contributed equally to this work.

Address all correspondence and requests for reprints to:

Jeong-Hoon Lee, MD, PhD
Associate Professor
Department of Internal Medicine

Seoul National University Hospital

101 Daehak-ro, Jongno-gu,

Seoul, 03080, Korea

Tel: 82-2-2072-2228, Fax: 82-2-743-6701

E-mail: pindra@empal.com or JHLeeMD@gmail.com

Contents

Supplementary Table S1. Baseline characteristics by subgroup according to FIB-4 scores at baseline. p. 1

Supplementary Table S2. Baseline characteristics by group after inverse probability weighting. p. 2

Supplementary Table S3. Baseline characteristics by group in patients with low-probability of significant fibrosis (FIB-4 <1.45). p. 3

Supplementary Table S4. Baseline characteristics by group in patients with intermediate-probability of significant fibrosis (FIB-4 1.45–3.25). p. 4

Supplementary Table S5. Baseline characteristics by group in patients with high-probability of significant fibrosis (FIB-4 ≥3.25). p. 5

Supplementary Table S6. Baseline characteristics by group in patients with low-probability of significant fibrosis (FIB-4 <1.45) after inverse probability weighting. p. 6

Supplementary Table S7. Baseline characteristics by group in patients with intermediate-probability of significant fibrosis (FIB-4 1.45–3.25) after inverse probability weighting. p. 7

Supplementary Table S8. Baseline characteristics by group in patients with high-probability of significant fibrosis (FIB-4 ≥3.25) after inverse probability weighting p. 8

Supplementary Table S9. Univariable and multivariable analysis of the clinical factors predictive of HCC development and all-cause mortality after inverse probability weighting. p. 9

Supplementary Table S10. Association between SVR and the risk of HCC among subgroups according to the probabilities of significant fibrosis after inverse probability weighting. p. 10

Supplementary Table S11. Baseline characteristics according to type of antiviral treatment regimen in patients achieving SVR. p. 11

Supplementary Table S12. Baseline characteristics according to type of antiviral treatment regimen in patients achieving SVR after inverse probability weighting. p. 12

Supplementary Fig. S1. Incidence of HCC and all-cause mortality by fibrosis stage assessed using FIB-4 scores. p. 13

Supplementary Fig. S2. Incidence of HCC by group after inverse probability weighting. p. 14

Supplementary Fig. S3. Incidence of all-cause mortality by group after inverse probability weighting. p. 15

Supplementary Fig. S4. Incidence of HCC and all-cause mortality by type of antiviral treatment regimen in patients achieving SVR. p. 16

Supplementary Fig. S5. Flowchart of identification and inclusion of the study subjects. p. 17

Supplementary Table S1. Baseline characteristics by subgroup according to FIB-4 scores at baseline

Characteristics	Overall (*N*=1196)	FIB-4 <1.45 (*n*=258)	FIB-4 1.45–3.25 (*n*=492)	FIB-4 ≥3.25 (*n*=446)	*P* value
Age, median (IQR), y	58 (51–67)	48 (39–54)	58 (53–65)	65 (57–71)	<.001
Male	485 (40.6)	111 (43)	212 (43.1)	162 (36.3)	.07
Genotype (*n*=1176)					.04
1	648 (55.1)	252 (50.6)	70 (55.4)	326 (57.3)	
2	512 (43.5)	184 (46.6)	46 (42.9)	282 (42.4)	
Other	16 (1.4)	6 (2.8)	4 (1.7)	6 (0.2)	
Laboratory data, median (IQR)					
Platelet count, $\times 10^9$/L	166 (128–208)	231 (201–264)	179 (152–205)	117 (89–147)	<.001
Albumin, g/dL (*n*=1195)	4.2 (4.0–4.4)	4.3 (4.2–4.5)	4.2 (4.1–4.4)	4.0 (3.7–4.2)	<.001
Total bilirubin, mg/dL (*n*=1195)	0.8 (0.6–1.1)	0.7 (0.6–1)	0.8 (0.7–1)	0.9 (0.7–1.2)	<.001
ALT, IU/L	52 (28–97)	42 (27–73)	56 (30–98)	58 (26–110)	.056
AST, IU/L	53 (33–82)	30 (22–41)	47 (32–71)	78 (54–116)	<.001
GGT, U/L (*n*=950)	42 (24–79)	29 (17–63)	36 (21–72)	52 (32–89)	.001
International normalised ratio (*n*=947)	1.03 (0.98–1.09)	0.99 (0.95–1.05)	1.01 (0.97–1.07)	1.07 (1.02–1.13)	<.001
AFP, ng/mL (*n*=1168)	5.0 (3.3–9.2)	3.8 (2.8–5.0)	3.8 (2.8–5.0)	5.0 (3.0–6.9)	<.001
HCV RNA, $\log_{10}$ IU/mL (*n*=1113)	6.04 (5.37–6.45)	6.13 (5.48–6.48)	6.13 (5.48–6.48)	6.09 (5.34–6.52)	.88
APRI					<.001
<0.5	357 (29.8)	199 (77.1)	148 (30.1)	10 (2.2)	
≥0.5 to <1.5	519 (43.4)	54 (20.9)	297 (60.4)	168 (37.7)	
≥1.5	320 (26.8)	5 (1.9)	47 (9.6)	268 (60.1)	
Diabetes mellitus	180 (15.1)	35 (13.6)	70 (14.2)	75 (16.8)	.41
Hypertension	219 (18.3)	29 (11.2)	87 (17.7)	103 (23.1)	<.001

Unless otherwise indicated, data are given as number (%) of patients.

Supplementary Table S2. Baseline characteristics by group after inverse probability weighting

Characteristics	Overall (*N*=767)	Untreated (*n*=296)	Treated without SVR (*n*=93)	Treated with SVR (*n*=378)	*P* value
Age, median (IQR), y	58 (51–66)	58 (52–66)	60 (53–65)	58 (50–67)	.94
Male	312 (40.6)	121 (40.6)	37 (40.5)	155 (40.5)	1.00
Genotype					1.00
1	412 (53.5)	158 (53.2)	47 (52.1)	206 (54)	
2	345 (44.8)	134 (45)	42 (46)	169 (44.4)	
Other	13 (1.7)	5 (1.8)	2 (1.9)	6 (1.6)	
Laboratory data, median (IQR)					
Platelet count, $\times 10^9$/L	166 (128–206)	171 (128–208)	158 (125–216)	165 (128–202)	.90
Albumin, g/dL	4.2 (4.0–4.4)	4.2 (4.0–4.4)	4.1 (4.0–4.4)	4.2 (4.0–4.4)	.85
Total bilirubin, mg/dL	0.8 (0.7–1.1)	0.8 (0.7–1.1)	0.9 (0.6–1.1)	0.8 (0.7–1.1)	.89
ALT, IU/L	59 (30–107)	53 (28–97)	62 (33–114)	65 (31–110)	.47
AST, IU/L	57 (35–91)	54 (34–87)	63 (35–91)	58 (37–94)	.53
GGT, U/L	43 (24–78)	43 (24–80)	45 (29–71)	41 (24–80)	.93
International normalised ratio	1.03 (0.98–1.09)	1.03 (0.98–1.09)	1.03 (0.99–1.12)	1.03 (0.98–1.09)	.76
APRI					.61
<0.5	206 (26.8)	85 (28.5)	22 (23.7)	100 (26.2)	
≥0.5 to <1.5	325 (42.2)	118 (39.6)	45 (49.8)	162 (42.5)	
≥1.5	238 (31)	95 (32)	24 (26.5)	119 (31.2)	
FIB-4					.98
<1.45	149 (19.3)	56 (18.9)	19 (21)	73 (19.2)	
≥1.45 to <3.25	322 (41.8)	127 (42.8)	35 (39)	159 (41.6)	
≥3.25	300 (38.9)	114 (38.3)	36 (40)	150 (39.2)	
Diabetes mellitus	114 (14.8)	43 (14.5)	15 (16.3)	56 (14.7)	.92
Hypertension	130 (16.8)	50 (16.8)	16 (17.1)	64 (16.8)	1.00

Unless otherwise indicated, data are given as number (%) of patients.

Supplementary Table S3. Baseline characteristics by group in patients with low-probability of significant fibrosis (FIB-4 <1.45)

Characteristics	Overall (*N*=258)	Untreated (*n*=108)	Treated without SVR (*n*=26)	Treated with SVR (*n*=124)	*P* value
Age, median (IQR), y	48 (39–54)	49 (40–55)	44 (39–51)	47 (39–53)	.52
Male	111 (43.0)	42 (38.9)	17 (65.4)	52 (41.9)	.05
Genotype (*n*=251)					.19
1	127 (50.6)	60 (57.7)	10 (40)	57 (46.7)	
2	117 (46.6)	42 (40.4)	13 (52)	62 (50.8)	
Other	7 (2.8)	2 (1.9)	2 (8)	3 (2.5)	
Laboratory data, median (IQR)					
Platelet count, $\times 10^9$/L	231 (201–264)	236 (203–266)	232 (209–247)	223 (197–264)	.30
Albumin, g/dL	4.3 (4.2–4.5)	4.3 (4.2–4.5)	4.5 (4.3–4.7)	4.4 (4.2–4.5)	.08
Total bilirubin, mg/dL	0.7 (0.6–1)	0.7 (0.6–1)	0.7 (0.6–0.9)	0.8 (0.6–0.9)	.54
ALT, IU/L	42 (27–72.5)	33 (24–58)	52 (45–83)	29 (44–88)	.14
AST, IU/L	30 (22–41)	28 (22–38)	33 (27–50)	34 (24–46)	.04
GGT, U/L (*n*=197)	29 (17–63)	27 (17–67)	57 (27–83)	26 (17–48)	.25
International normalised ratio (*n*=197)	0.99 (0.95–1.05)	1.01 (0.96–1.06)	0.97 (0.94–1.03)	0.98 (0.95–1.04)	.22
AFP, ng/mL (*n*=252)	3.8 (2.8–5.0)	3.6 (2.6–5.0)	4.9 (3.4–5.5)	4.0 (2.8–5)	.66
HCV RNA, $\log_{10}$ IU/mL (*n*=241)	6.13 (5.48–6.48)	6.01 (5.34–6.42)	6.39 (5.85–6.64)	6.16 (5.46–6.46)	.04
APRI					.05
<0.5	199 (77.1)	91 (84.3)	16 (61.5)	92 (74.2)	
≥0.5 to <1.5	54 (20.9)	16 (14.8)	10 (38.5)	28 (22.6)	
≥1.5	5 (1.9)	1 (0.9)	0 (0)	4 (3.2)	
Diabetes mellitus	35 (13.6)	19 (17.6)	4 (15.4)	12 (9.7)	.21
Hypertension	29 (11.2)	17 (15.7)	3 (11.5)	9 (7.3)	.13

Unless otherwise indicated, data are given as number (%) of patients.

Supplementary Table S4. Baseline characteristics by group in patients with intermediate-probability of significant fibrosis (FIB-4 1.45–3.25)

Characteristics	Overall<br>(*N*=492)	Untreated<br>(*n*=185)	Treated without SVR<br>(*n*=47)	Treated with SVR<br>(*n*=260)	*P* value
Age, median (IQR), y	58 (53–65)	59 (53–65)	59 (53–63)	57 (52–65)	.86
Male	212 (43.1)	85 (45.9)	18 (38.3)	109 (41.9)	.55
Genotype (*n*=482)					.24
1	267 (55.4)	100 (56.5)	29 (61.7)	138 (53.5)	
2	207 (42.9)	73 (41.2)	16 (34)	118 (45.7)	
Other	8 (1.7)	4 (2.3)	2 (4.3)	2 (0.8)	
Laboratory data, median (IQR)					
Platelet count, $\times 10^9$/L	179 (152–205)	180 (154–208)	167 (140–201)	179 (152–202)	.49
Albumin, g/dL	4.2 (4.1–4.4)	4.2 (4.0–4.4)	4.2 (4.0–4.3)	4.2 (4.1–4.4)	.09
Total bilirubin, mg/dL	0.8 (0.7–1.0)	0.8 (0.6–1.0)	0.9 (0.6–1.1)	0.8 (0.7–1.0)	.61
ALT, IU/L	56 (30–98)	50 (30–85)	56 (29–93)	64 (31–106)	.06
AST, IU/L	47 (32–71)	45 (33–65)	46 (29–71)	50 (32–78)	.12
GGT, U/L (*n*=391)	36 (21–72)	33 (21–68)	39 (22–71)	36 (22–75)	1.00
International normalised ratio (*n*=392)	1.01 (0.97–1.07)	1.01 (0.98–1.09)	1.03 (0.99–1.08)	1.01 (0.96–1.06)	.15
AFP, ng/mL (*n*=485)	5.0 (3.0–6.9)	4.7 (3.0–7.0)	4.9 (3.1–6.3)	5.0 (3.2–6.7)	.34
HCV RNA, $\log_{10}$ IU/mL (*n*=456)	6.09 (5.34–6.52)	5.99 (5.29–6.41)	6.43 (6.1–6.66)	6.06 (5.31–6.5)	<.001
APRI					.38
<0.5	148 (30.1)	56 (30.3)	14 (29.8)	78 (30)	
≥0.5 to <1.5	297 (60.4)	115 (62.2)	31 (66)	151 (58.1)	
≥1.5	47 (9.6)	14 (7.6)	2 (4.3)	31 (11.9)	
Diabetes mellitus	70 (14.2)	28 (15.1)	7 (14.9)	35 (13.5)	.88
Hypertension	87 (17.7)	27 (14.6)	10 (21.3)	50 (19.2)	.36

Unless otherwise indicated, data are given as number (%) of patients.

Supplementary Table S5. Baseline characteristics by group in patients with high-probability of significant fibrosis (FIB-4 ≥3.25)

Characteristics	Overall (*N*=446)	Untreated (*n*=163)	Treated without SVR (*n*=49)	Treated with SVR (*n*=234)	*P* value
Age, median (IQR), y	65 (57–71)	66 (59–71)	63 (56–67)	66 (57–72)	.06
Male	162 (36.3)	62 (38)	17 (34.7)	83 (35.5)	.85
Genotype (*n*=443)					.73
1	254 (57.3)	92 (57.1)	31 (64.6)	131 (56)	
2	188 (42.4)	69 (42.9)	17 (35.4)	102 (43.6)	
Other	1 (0.2)	0 (0)	0 (0)	1 (0.4)	
Laboratory data, median (IQR)					
Platelet count, $\times 10^9$/L	117 (89–147)	116 (86–147)	112 (92–131)	122 (92–151)	.21
Albumin, g/dL (*n*=445)	4.0 (3.7–4.2)	4 (3.8–4.2)	4 (3.7–4.1)	4 (3.7–4.2)	.67
Total bilirubin, mg/dL (*n*=445)	0.9 (0.7–1.2)	0.9 (0.7–1.2)	0.9 (0.8–1.2)	0.9 (0.7–1.2)	.95
ALT, IU/L	58 (26–110)	48 (26–92)	58 (27–111)	67 (27–126)	.15
AST, IU/L	78 (54–116)	77 (52–110.5)	79 (54–98)	79 (55–122)	.13
GGT, U/L (*n*=362)	52 (32–89)	54 (36–98)	52 (33–82)	51 (30–86)	.69
International normalised ratio (*n*=358)	1.07 (1.02–1.13)	1.07 (1.02–1.13)	1.09 (1.02–1.18)	1.07 (1.01–1.11)	.38
AFP, ng/mL (*n*=431)	8.5 (5.0–16.5)	8.6 (5.0–17.6)	9.0 (6.0–30.1)	7.7 (5.0–15.2)	.11
HCV RNA, $\log_{10}$ IU/mL (*n*=416)	5.99 (5.37–6.34)	5.88 (5.28–6.24)	6.39 (6.02–6.6)	5.93 (5.35–6.31)	<.001
APRI					.76
<0.5	10 (2.2)	3 (1.8)	0 (0)	7 (3)	
≥0.5 to <1.5	168 (37.7)	62 (38)	19 (38.8)	87 (37.2)	
≥1.5	268 (60.1)	98 (60.1)	30 (61.2)	140 (59.8)	
Diabetes mellitus	75 (16.8)	27 (16.6)	10 (20.4)	38 (16.2)	.77
Hypertension	103 (23.1)	39 (23.9)	9 (18.4)	55 (23.5)	.70

Unless otherwise indicated, data are given as number (%) of patients.

Supplementary Table S6. Baseline characteristics by group in patients with low-probability of significant fibrosis (FIB-4 <1.45) after inverse probability weighting

Characteristics	Overall (*N*=148)	Untreated (*n*=57)	Treated without SVR (*n*=19)	Treated with SVR (*n*=72)	*P* value
Age, median (IQR), y	48 (40–53)	48 (42–54)	49 (42–53)	47 (38–53)	.53
Male	65 (43.9)	21 (36.7)	13 (66.4)	32 (43.6)	.09
Genotype					.75
1	74 (49.7)	30 (54)	7 (37.1)	36 (49.6)	
2	69 (46.3)	24 (42.9)	11 (59)	33 (45.6)	
Other	6 (4.0)	2 (3.1)	1 (3.8)	3 (4.8)	
Laboratory data, median (IQR)					
Platelet count, $\times 10^9$/L	234 (201–265)	236 (200–266)	235 (218–254)	226 (197–278)	.69
Albumin, g/dL	4.4 (4.2–4.6)	4.4 (4.3–4.6)	4.6 (4.3–4.8)	4.4 (4.2–4.5)	.24
Total bilirubin, mg/dL	0.7 (0.6–1.0)	0.8 (0.6–1.1)	0.6 (0.6–0.8)	0.8 (0.6–1)	.25
ALT, IU/L	42 (27–76)	32 (25–61)	49 (44–86)	44 (29–97)	.66
AST, IU/L	29 (23–43)	28 (22–40)	29 (24–44)	34 (25–47)	.64
GGT, U/L	28 (18–61)	25 (17–62)	59 (29–71)	26 (17–49)	.99
International normalised ratio	0.99 (0.95–1.05)	1 (0.95–1.06)	1 (0.95–1.03)	0.98 (0.96–1.04)	.61
APRI					.51
<0.5	118 (79.2)	47 (84.2)	13 (70.4)	57 (77.6)	
≥0.5 to <1.5	27 (18.4)	7 (12.1)	6 (29.6)	15 (20.4)	
≥1.5	4 (2.4)	2 (3.8)	0 (0)	1 (1.9)	
Diabetes mellitus	19 (13)	7 (12.4)	4 (22.5)	8 (11)	.43
Hypertension	18 (12)	8 (15.1)	3 (17.6)	6 (8.2)	.39

Unless otherwise indicated, data are given as number (%) of patients.

Supplementary Table S7. Baseline characteristics by group in patients with intermediate-probability of significant fibrosis (FIB-4 1.45–3.25) after inverse probability weighting

Characteristics	Overall (*N*=321)	Untreated (*n*=116)	Treated without SVR (*n*=37)	Treated with SVR (*n*=168)	*P* value
Age, median (IQR), y	58 (53–64)	58 (53–64)	60 (57–65)	58 (52–65)	.43
Male	136 (42.2)	56 (44)	12 (33.7)	68 (42.7)	.59
Genotype					.89
1	174 (54.2)	68 (53.6)	19 (54.2)	87 (54.6)	
2	141 (43.9)	55 (43.5)	15 (42.9)	70 (44.3)	
Other	6 (2)	4 (2.9)	1 (2.9)	2 (1.1)	
Laboratory data, median (IQR)					
Platelet count, $\times 10^9$/L	180 (151–206)	180 (150–208)	179 (156–212)	181 (152–201)	.41
Albumin, g/dL	4.2 (4.1–4.4)	4.3 (4–4.4)	4.3 (4–4.3)	4.2 (4.1–4.4)	.38
Total bilirubin, mg/dL	0.8 (0.7–1.0)	0.8 (0.6–1)	0.9 (0.7–1.1)	0.8 (0.7–1)	.92
ALT, IU/L	60 (33–107)	57 (36–98)	63 (39–103)	67 (33–109)	.60
AST, IU/L	49 (34–73)	45 (34–69)	53 (35–73)	52 (33–78)	.73
GGT, U/L	38 (22–74)	38 (24–73)	48 (22–69)	38 (22–78)	.82
International normalised ratio	1.01 (0.97–1.07)	1 (0.97–1.07)	1.03 (0.99–1.07)	1.01 (0.97–1.07)	.93
APRI					.21
<0.5	87 (27.1)	36 (28.2)	8 (22.9)	43 (27.2)	
≥0.5 to <1.5	197 (61.3)	75 (58.9)	27 (77.1)	95 (59.6)	
≥1.5	37 (11.6)	16 (12.9)	0 (0)	21 (13.1)	
Diabetes mellitus	47 (14.6)	18 (14.4)	4 (10.8)	25 (15.6)	.76
Hypertension	49 (15.2)	16 (12.7)	6 (17.6)	26 (16.7)	.61

Unless otherwise indicated, data are given as number (%) of patients.

Supplementary Table S8. Baseline characteristics by group in patients with high-probability of significant fibrosis (FIB-4 ≥3.25) after inverse probability weighting

**Characteristics**	**Overall** (***N*=298**)	**Untreated** (***n*=123**)	**Treated without SVR** (***n*=37**)	**Treated with SVR** (***n*=138**)	***P* value**
Age, median (IQR), y	64 (57–70)	64 (57–71)	63 (57–67)	64 (56–71)	.54
Male	111 (37.1)	44 (38.9)	12 (33.4)	55 (36.6)	.85
Genotype					.86
1	164 (54.6)	60 (52.5)	21 (57.9)	83 (55.5)	
2	135 (45)	54 (47.5)	15 (42.1)	65 (43.8)	
Other	1 (0.4)	0 (0)	0 (0)	1 (0.7)	
Laboratory data, median (IQR)					
Platelet count, ×10^9/L	122 (88–151)	120 (85–157)	120 (98–143)	123 (86–149)	.77
Albumin, g/dL	4.0 (3.8–4.2)	4.0 (3.8–4.2)	4.0 (3.9–4.1)	4.0 (3.7–4.3)	.72
Total bilirubin, mg/dL	0.9 (0.7–1.2)	0.9 (0.7–1.2)	0.9 (0.8–1.3)	0.9 (0.7–1.3)	.98
ALT, IU/L	68 (29–122)	59 (28–120)	84 (30–118)	69 (35–130)	.46
AST, IU/L	82 (59–121)	81 (57–121)	91 (65–120)	82 (62–122)	.47
GGT, U/L	51 (34–92)	51 (37–99)	44 (33–78)	52 (31–90)	.81
International normalised ratio	1.07 (1.02–1.14)	1.07 (1.01–1.13)	1.09 (1.02–1.17)	1.07 (1.02–1.14)	.59
APRI					.65
<0.5	1 (0.5)	1 (1.3)	0 (0)	0 (0)	
≥0.5 to <1.5	101 (33.6)	36 (31.5)	12 (33.8)	53 (96.9)	
≥1.5	198 (65.9)	77 (67.2)	24 (66.2)	35 (64.8)	
Diabetes mellitus	48 (16)	18 (15.6)	7 (18.3)	23 (15.6)	.93
Hypertension	63 (20.9)	25 (22.2)	6 (16.2)	32 (21.2)	.76

Unless otherwise indicated, data are given as number (%) of patients.

Supplementary Table S9. Univariable and multivariable analysis of the clinical factors predictive of HCC development and all-cause mortality after inverse probability weighting

	HCC development				All-cause mortality			
	Univariable analysis		Multivariable analysis		Univariable analysis		Multivariable analysis	
	**Hazard ratio (95% CI)**	***P* value**	**Adjusted hazard ratio (95% CI)**	***P* value**	**Hazard ratio (95% CI)**	***P* value**	**Adjusted hazard ratio (95% CI)**	***P* value**
Age, y	1.071 (1.043–1.099)	.04			1.061 (1.003–1.124)	.04	1.053 (1.013–1.094)	.009
Male	2.408 (1.287–4.506)	.01	2.351 (1.279–4.32)	.006	4.251 (1.615–11.186)	.003	4.762 (2.096–10.821)	<.001
Genotype		.66				.01		<.001
1	1 [Reference]				1 [Reference]		1 [Reference]	
2	0.765 (0.411–1.424)	.40			1.173 (0.486–2.831)	.72	1.062 (0.426–2.648)	.9
Other	0.628 (0.078–5.087)	.66			6.61 (1.877–23.282)	.003	17.633 (5.703–54.515)	<.001
Platelet count, $\times 10^9$/L	0.984 (0.978–0.99)	<.001			0.987 (0.979–0.996)	.003		
Albumin, g/dL	0.199 (0.103–0.383)	<.001	0.408 (0.189–0.88)	.02	0.167 (0.062–0.448)	<.001	0.345 (0.136–0.875)	.03
Total bilirubin, mg/dL	2.616 (1.498–4.569)	<.001	2.004 (1.093–3.672)	.03	3.485 (1.56–7.784)	.002		
ALT, IU/L	0.998 (0.995–1.002)	.37			0.998 (0.993–1.002)	.28		
AST, IU/L	1.002 (0.999–1.005)	.14			1.001 (0.996–1.006)	.73		
GGT, IU/L	1.003 (1–1.006)	.03	1.002 (0.998–1.006)	.25	1.001 (0.999–1.004)	.34		
INR	2.446 (0.55–10.883)	.24			2.846 (0.587–13.788)	.19		
APRI		.001				.19		
<0.5	1 [Reference]				1 [Reference]			
≥0.5 to <1.5	2.145 (0.693–6.639)	.19			1.334 (0.345–5.163)	.68		
≥1.5	5.26 (1.803–15.345)	.002			2.619 (0.733–9.356)	.14		
FIB-4		<.001		.008				
<1.45	1 [Reference]		1 [Reference]					
≥1.45 to <3.25	4.685 (0.586–37.441)	.15	2.963 (0.359–24.465)	.31				
≥3.25	15.191 (2.038–113.251)	.008	7.647 (1.008–57.994)	.049				
Diabetes mellitus								
No	1 [Reference]				1 [Reference]			
Yes	1.61 (0.79–3.282)	.19			2.09 (0.81–5.391)	.13		
Hypertension								
No	1 [Reference]				1 [Reference]			
Yes	1.514 (0.708–3.237)	.28			0.996 (0.316–3.144)	.99		
Treatment		.003		.001		<.001		.001
Untreated	1 [Reference]		1 [Reference]		1 [Reference]		1 [Reference]	
Treated wihout SVR	0.609 (0.267–1.385)	.24	0.794 (0.346–1.826)	.59	0.083 (0.011–0.63)	.02	0.124 (0.016–0.937)	.04
Treated wih SVR	0.229 (0.098–0.533)	<.001	0.226 (0.099–0.521)	<.001	0.128 (0.038–0.433)	.001	0.171 (0.051–0.577)	.004

P values were determined using Cox proportional hazards regression models. *P* < 0.05 indicated a significant difference.

Supplementary Table S10. Association between SVR and the risk of HCC among subgroups according to the probabilities of significant fibrosis after inverse probability weighting

	HCC development		All-cause mortality	
	**Adjusted hazard ratio (95% CI)**	***P* value**	**Adjusted hazard ratio (95% CI)**	***P* value**
FIB-4<1.45				
Untreated	1 [Reference]		–	
Treated without SVR	2.947 (0.008–1153.611)	.72	–	
Treated with SVR	2.508 (0.020–312.583)	.71	–	
FIB-4 ≥1.45 to <3.25				
Untreated	1 [Reference]		1 [Reference]	
Treated without SVR	0.173 (0.018–1.661)	.13	0.384 (0.028–5.345)	.48
Treated with SVR	0.033 (0.002–0.706)	.03	0.450 (0.100–2.026)	.30
FIB-4 ≥3.25				
Untreated	1 [Reference]		1 [Reference]	
Treated without SVR	1.294 (0.531–3.153)	.57	0.073 (0.004–1.494)	.09
Treated with SVR	0.305 (0.126–0.739)	.009	0.033 (0.002–0.683)	.03

P values were determined using multivariable Cox proportional hazards regression models.

Supplementary Table S11. Baseline characteristics according to type of antiviral treatment regimen in patients achieving SVR

Characteristics	Overall (*N*=622)	IFN-based therapy (*n*=314)	DAA therapy (*n*=308)	*P* value
Age, median (IQR), y	58 (50–68)	54 (47–60)	66 (57–72)	<.001
Male	246 (39.5)	140 (44.6)	106 (34.4)	.01
Genotype (*n*=618)				<.001
1	326 (52.8)	94 (30.3)	232 (75.3)	
2	286 (46.3)	210 (67.7)	76 (24.7)	
Other	6 (1)	6 (1.9)	0 (0)	
Laboratory data, median (IQR)				
Platelet count, $\times 10^9$/L (*n*=618)	165 (128–203)	177 (139–211)	156 (118–194)	<.001
Albumin, g/dL	4.2 (4–4.4)	4.3 (4.1–4.4)	4.1 (3.8–4.3)	<.001
Total bilirubin, mg/dL	0.8 (0.7–1.1)	0.8 (0.7–1.0)	0.8 (0.7–1.1)	.02
ALT, IU/L	59 (29–107)	73 (36–137)	45 (25–82)	<.001
AST, IU/L	56 (34–92)	61 (34–103)	52 (33–79)	.001
GGT, U/L (*n*=483)	40 (23–77)	48 (25–90)	35 (20–60)	<.001
International normalised ratio (*n*=474)	1.02 (0.97–1.08)	1.03 (0.97–1.08)	1.01 (0.97–1.08)	.11
AFP, ng/mL (*n*=605)	5.0 (3.4–8.6)	5.0 (3.2–9)	5.0 (3.7–8.2)	.66
HCV RNA, $\log_{10}$ IU/mL (*n*=586)	6.03 (5.34–6.43)	5.87 (5.02–6.38)	6.1 (5.68–6.51)	<.001
APRI (*n*=618)				.81
<0.5	177 (28.6)	89 (28.7)	88 (28.6)	
≥0.5 to <1.5	266 (43)	130 (41.9)	136 (44.2)	
≥1.5	175 (28.3)	91 (29.4)	84 (27.3)	
FIB-4 (*n*=618)				<.001
<1.45	124 (20.1)	90 (29)	34 (11)	
≥1.45 to <3.25	260 (42.1)	128 (41.3)	132 (42.9)	
≥3.25	234 (37.9)	92 (29.7)	142 (46.1)	
Diabetes mellitus	85 (13.7)	40 (12.7)	45 (14.6)	.50
Hypertension	115 (18.5)	40 (12.7)	75 (24.4)	<.001

Unless otherwise indicated, data are given as number (%) of patients.

Supplementary Table S12. Baseline characteristics according to type of antiviral treatment regimen in patients achieving SVR after inverse probability weighting

Characteristics	Overall (*N*=378)	IFN-based therapy (*n*=222)	DAA therapy (*n*=156)	*P* value
Age, median (IQR), y	57 (50–66)	57 (50–66)	59 (49–68)	.93
Male	148 (41.2)	97 (44.3)	51 (36.4)	.30
Genotype				.45
1	187 (52.0)	110 (50)	77 (55.1)	
2	170 (47.3)	107 (48.9)	63 (44.9)	
Other	2 (0.7)	2 (1.1)	0 (0)	
Laboratory data, median (IQR)				
Platelet count, $\times 10^9$/L	166 (128–204)	167 (131–207)	163 (122–202)	.58
Albumin, g/dL	4.2 (4.0–4.4)	4.2 (4–4.4)	4.1 (4–4.3)	.23
Total bilirubin, mg/dL	0.8 (0.7–1.1)	0.8 (0.7–1.1)	0.8 (0.7–1.1)	.73
ALT, IU/L	69 (31–130)	66 (29–132)	74 (38–130)	.67
AST, IU/L	59 (40–105)	55 (40–101)	62 (41–108)	.50
GGT, U/L	41 (25–79)	41 (26–81)	43 (23–74)	.32
International normalised ratio	1.02 (0.97–1.07)	1.02 (0.97–1.07)	1.01 (0.98–1.08)	.98
APRI				.81
<0.5	89 (24.8)	58 (26.5)	31 (22.2)	
≥0.5 to <1.5	156 (43.5)	93 (42.6)	63 (44.9)	
≥1.5	114 (31.7)	68 (30.9)	46 (32.9)	
FIB-4				.44
<1.45	74 (20.5)	52 (23.6)	22 (15.6)	
≥1.45 to <3.25	137 (38.2)	76 (34.7)	61 (43.6)	
≥3.25	148 (41.3)	91 (41.7)	57 (40.8)	
Diabetes mellitus	55 (15.3)	34 (15.6)	21 (14.7)	.86
Hypertension	67 (18.8)	41 (18.6)	27 (19.1)	.92

Unless otherwise indicated, data are given as number (%) of patients.

Supplementary Fig. S1. Incidence of HCC and all-cause mortality by fibrosis stage assessed using FIB-4 scores. (A) Incidence of HCC. (B) Incidence of all-cause mortality.

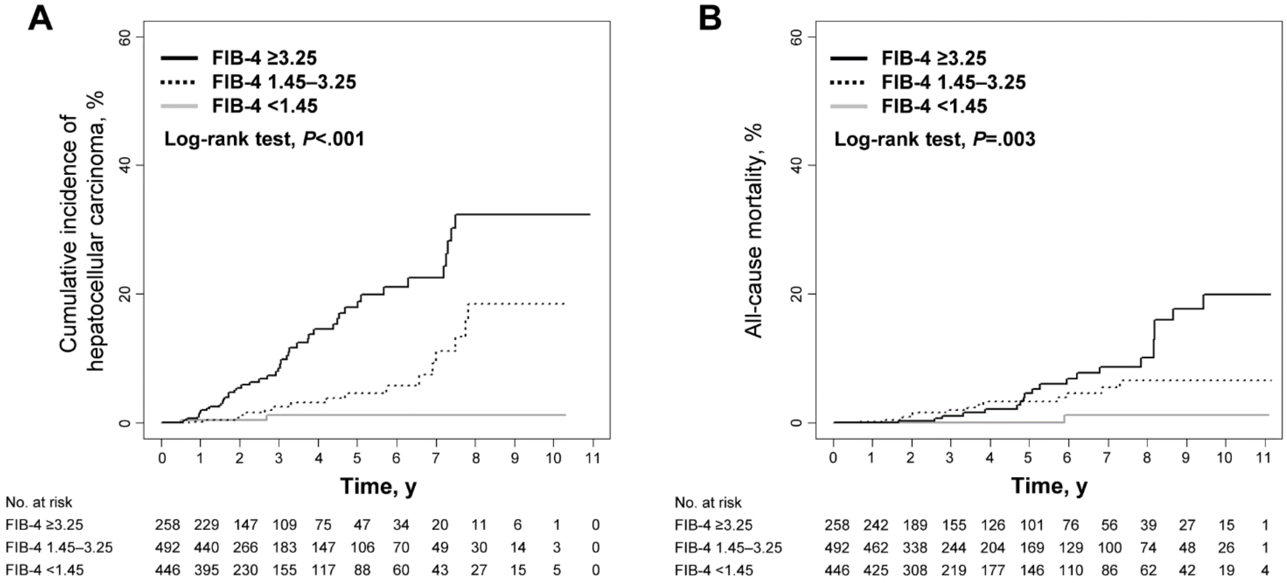

Supplementary Fig. S2. Incidence of HCC by group after inverse probability weighting. (A) In the entire study population. (B) In patients with low-probability of significant fibrosis (FIB-4 <1.45). (C) In patients with intermediate-probability of significant fibrosis (FIB-4 1.45–3.25). (D) In patients with high-probability of significant fibrosis (FIB-4 ≥3.25).

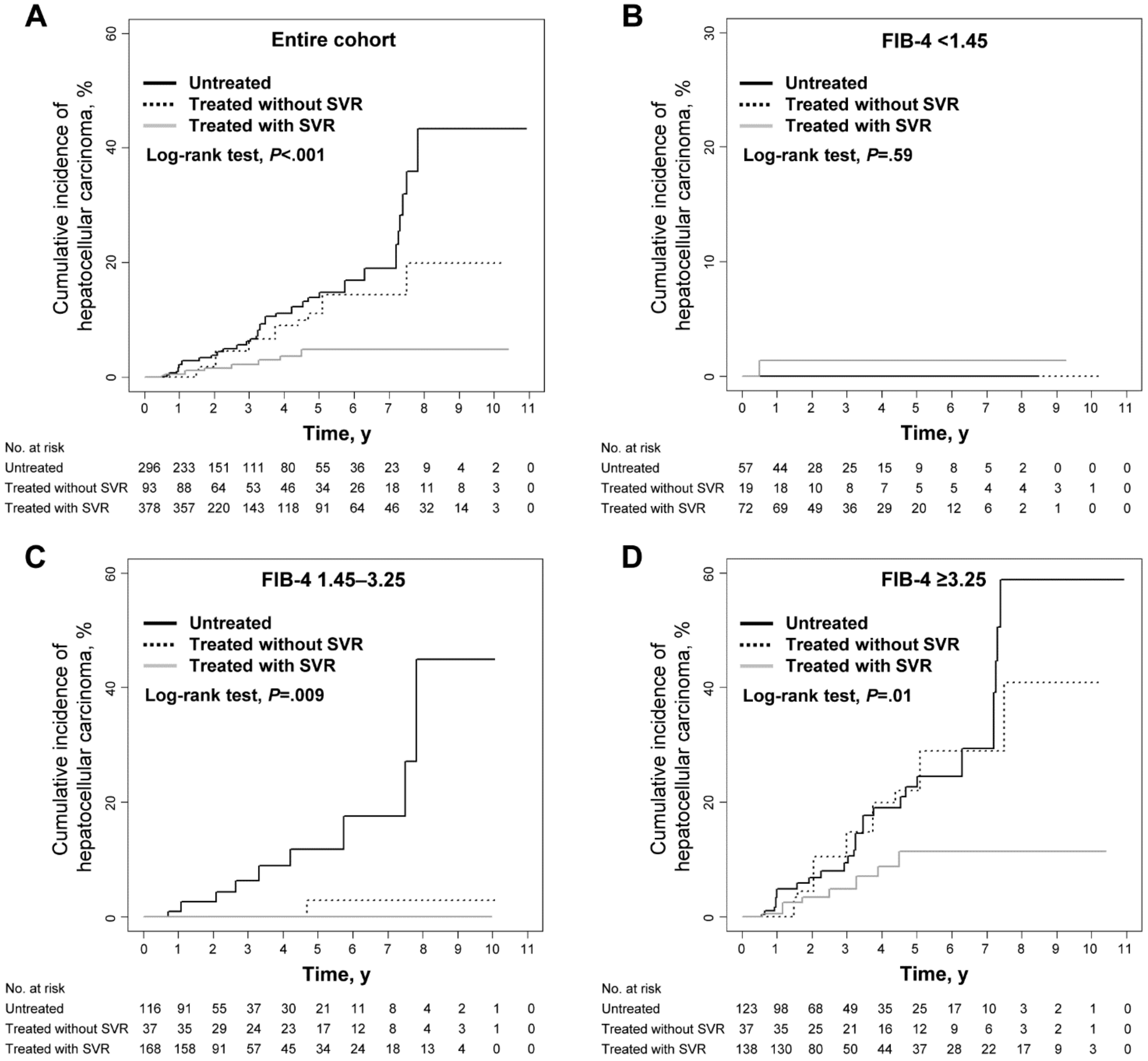

A

No. at risk	0	1	2	3	4	5	6	7	8	9	10	11
Untreated	296	233	151	111	80	55	36	23	9	4	2	0
Treated without SVR	93	88	64	53	46	34	26	18	11	8	3	0
Treated with SVR	378	357	220	143	118	91	64	46	32	14	3	0

B

No. at risk	0	1	2	3	4	5	6	7	8	9	10	11
Untreated	57	44	28	25	15	9	8	5	2	0	0	0
Treated without SVR	19	18	10	8	7	5	5	4	4	3	1	0
Treated with SVR	72	69	49	36	29	20	12	6	2	1	0	0

C

No. at risk	0	1	2	3	4	5	6	7	8	9	10	11
Untreated	116	91	55	37	30	21	11	8	4	2	1	0
Treated without SVR	37	35	29	24	23	17	12	8	4	3	1	0
Treated with SVR	168	158	91	57	45	34	24	18	13	4	0	0

D

No. at risk	0	1	2	3	4	5	6	7	8	9	10	11
Untreated	123	98	68	49	35	25	17	10	3	2	1	0
Treated without SVR	37	35	25	21	16	12	9	6	3	2	1	0
Treated with SVR	138	130	80	50	44	37	28	22	17	9	3	0

Supplementary Fig. S3. Incidence of all-cause mortality by group after inverse probability weighting. (A) In the entire study population. (B) In patients with low-probability of significant fibrosis (FIB-4 <1.45). (C) In patients with intermediate-probability of significant fibrosis (FIB-4 1.45–3.25). (D) In patients with high-probability of significant fibrosis (FIB-4 ≥3.25).

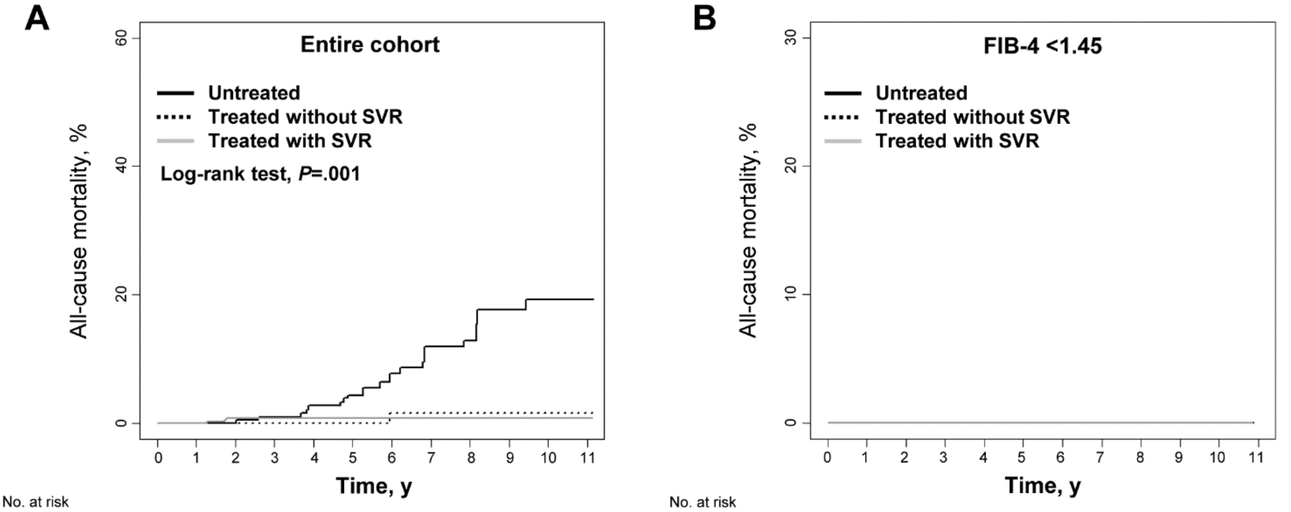

A

No. at risk	0	1	2	3	4	5	6	7	8	9	10	11
Untreated	296	259	208	172	140	118	88	68	48	30	16	1
Treated without SVR	93	92	82	73	67	56	40	32	22	18	12	1
Treated with SVR	378	371	268	184	163	145	116	87	63	43	19	3

B

No. at risk	0	1	2	3	4	5	6	7	8	9	10	11
Untreated	57	49	40	35	27	26	20	17	11	7	4	0
Treated without SVR	19	18	13	13	12	9	8	7	5	5	4	0
Treated with SVR	72	71	62	51	43	38	28	18	13	7	3	0

C

No. at risk	0	1	2	3	4	5	6	7	8	9	10	11
Untreated	116	99	77	60	50	41	29	22	15	11	6	1
Treated without SVR	37	37	34	28	26	23	16	13	7	4	3	0
Treated with SVR	168	164	111	74	66	58	47	34	26	19	9	0

D

No. at risk	0	1	2	3	4	5	6	7	8	9	10	11
Untreated	123	111	91	77	63	51	39	29	22	12	6	0
Treated without SVR	37	37	35	32	29	24	16	12	10	9	5	1
Treated with SVR	138	136	95	59	54	49	41	35	24	17	7	3

Supplementary Fig. S4. Incidence of HCC and all-cause mortality by type of antiviral treatment regimen in patients achieving SVR. (A) Incidence of HCC. (B) Incidence of all-cause mortality. (C) Incidence of HCC after inverse probability weighting. (D) Incidence of all-cause mortality after inverse probability weighting.

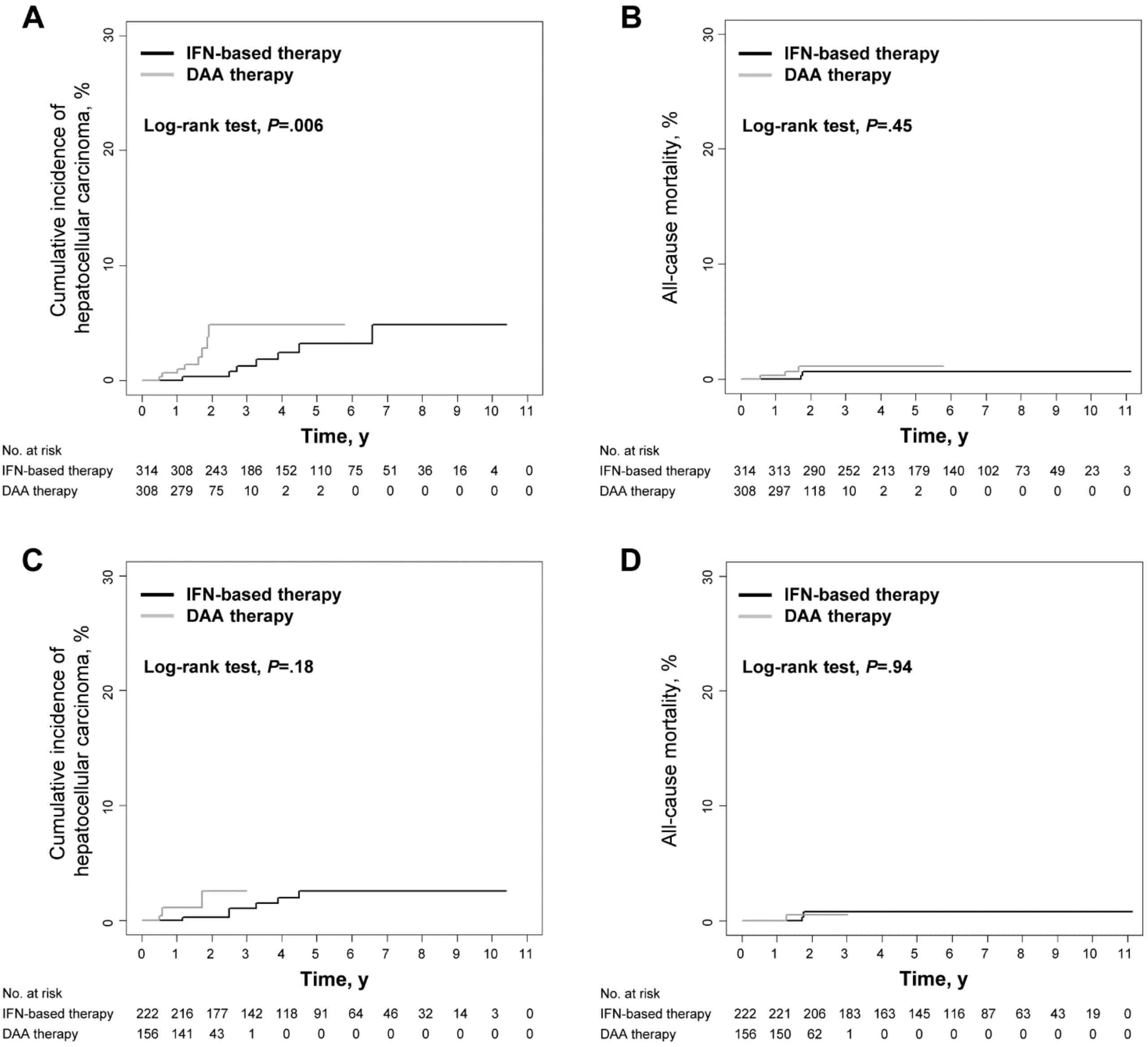

Supplementary Fig. S5. Flowchart of identification and inclusion of the study subjects.

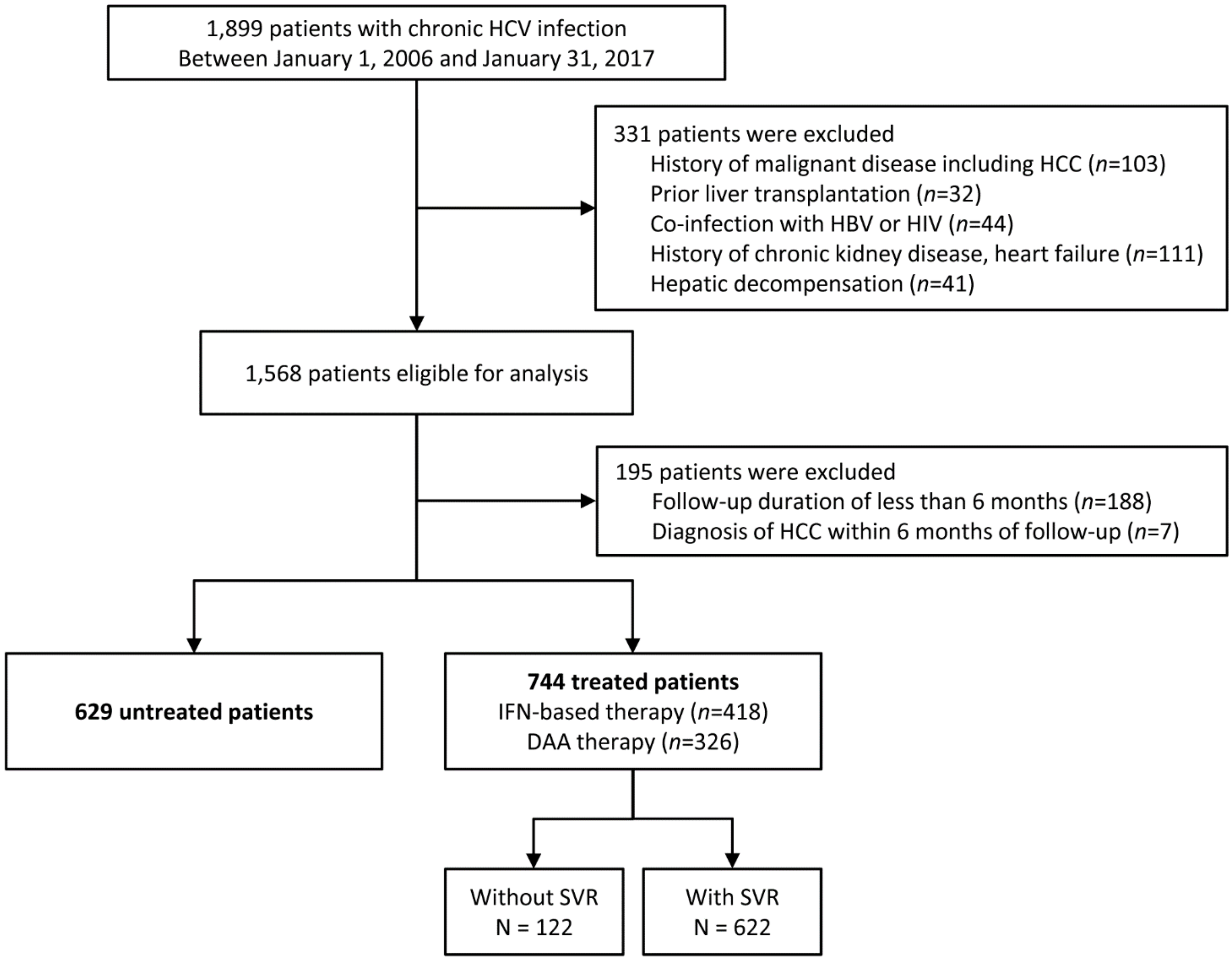

Supplement: Supplementary file 1 — Supplementary Material [file 41598_2018_31839_MOESM1_ESM.pdf]
